# Rock, scissors, paper: How RNA structure informs function

**DOI:** 10.1093/plcell/koad026

**Published:** 2023-02-07

**Authors:** Sarah M Assmann, Hong-Li Chou, Philip C Bevilacqua

**Affiliations:** Department of Biology, Pennsylvania State University, University Park, PA 16802, USA; Center for RNA Molecular Biology, Pennsylvania State University, University Park, PA 16802, USA; Department of Biology, Pennsylvania State University, University Park, PA 16802, USA; Center for RNA Molecular Biology, Pennsylvania State University, University Park, PA 16802, USA; Department of Chemistry, Pennsylvania State University, University Park, PA 16802, USA; Department of Biochemistry and Molecular Biology, Pennsylvania State University, University Park, PA 16802, USA

## Abstract

RNA can fold back on itself to adopt a wide range of structures. These range from relatively simple hairpins to intricate 3D folds and can be accompanied by regulatory interactions with both metabolites and macromolecules. The last 50 yr have witnessed elucidation of an astonishing array of RNA structures including transfer RNAs, ribozymes, riboswitches, the ribosome, the spliceosome, and most recently entire RNA structuromes. These advances in RNA structural biology have deepened insight into fundamental biological processes including gene editing, transcription, translation, and structure-based detection and response to temperature and other environmental signals. These discoveries reveal that RNA can be relatively static, like a rock; that it can have catalytic functions of cutting bonds, like scissors; and that it can adopt myriad functional shapes, like paper. We relate these extraordinary discoveries in the biology of RNA structure to the plant way of life. We trace plant-specific discovery of ribozymes and riboswitches, alternative splicing, organellar ribosomes, thermometers, whole-transcriptome structuromes and pan-structuromes, and conclude that plants have a special set of RNA structures that confer unique types of gene regulation. We finish with a consideration of future directions for the RNA structure–function field.

## Introduction

As in all living systems, RNA in plants has multifarious functions, the majority of which are predicated on the proper folding of the RNA. Like proteins, RNA possesses a structure that can be described at 5 different levels: primary, secondary, tertiary, quaternary, and quinary. The primary structure is the RNA sequence itself. Secondary structure describes the paired and unpaired elements of stems, loops, and bulges that arise as the single-stranded RNA molecule folds back on itself and interacts via hydrogen bonding and stacking. Tertiary or 3D structure typically compacts the RNA and is achieved by longer distance Watson–Crick and non-Watson–Crick interactions of elements within the pre-formed secondary structures. These interactions give rise to tertiary structural elements, including pseudoknots, which lock together 2 stem-loops (SLs) by base pairing and sugar-phosphate interactions, often in a so-called kissing interaction. Quaternary structure results from a folded RNA's interaction with other biopolymers such as proteins and other RNAs, and quinary structure (Monteith et al. 2015) arises from RNA's weak and nonspecific interaction with cellular metabolites such as osmolytes (Lambert and Draper 2007). An inspiring perspective on RNA structure has recently become available that touts the ubiquity, diversity, and dynamic nature of RNA structure (Vicens and Kieft 2022).

A given protein typically has a more limited number of closely related conformations it can take on and remain functional, and this “rock-like” property largely holds for some highly structured RNAs, such as the ribosome and transfer RNAs (tRNAs). However, unlike most proteins, many RNAs populate 2 or more distinct conformations with different functions (Tomezsko et al. 2020), while for others, particularly mRNAs, an individual sequence can attain a vast ensemble of secondary and tertiary structures, thus being akin to origami or “paper-like” in their attributes, all of which point to the dynamic nature of RNA (Mathews 2004; Andrews et al. 2018; Spasic et al. 2018). With the discovery of self-cleaving ribozymes in the 1980s, it additionally was found that some RNAs function as molecular “scissors.” In this review, we discuss different classes of RNAs, how they fall into the “rock,” “paper,” and “scissors” categories, and their associated plant biology.

**Figure 1.**
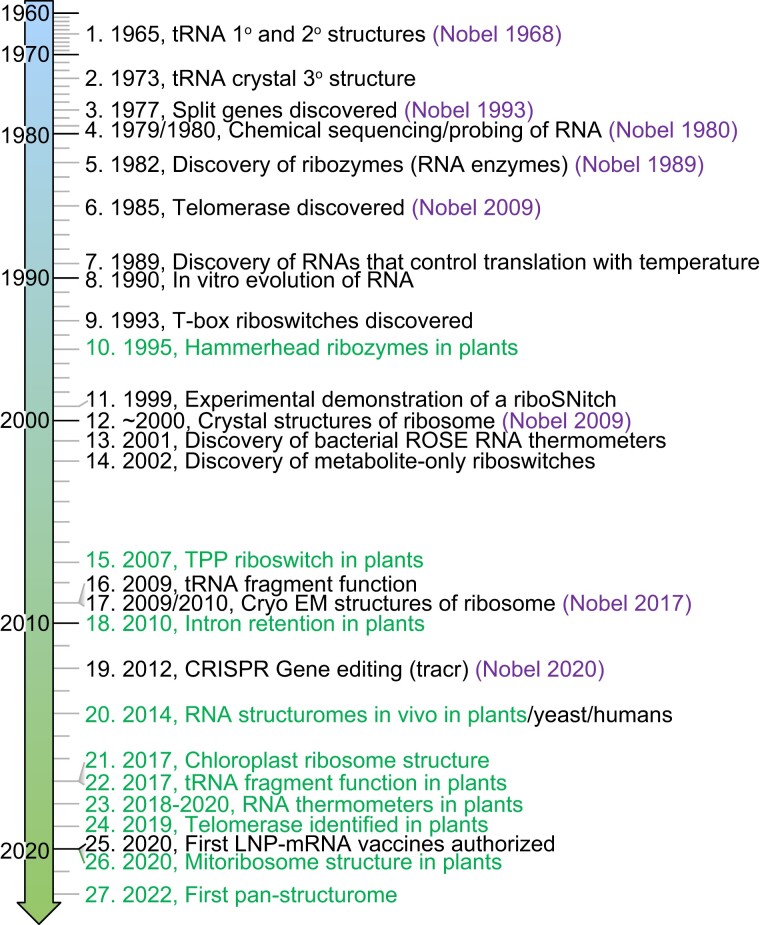
Timeline for seminal discoveries in RNA structural biology. Related Nobel Prizes are in purple font and important plant discoveries are in green font. LNP, lipid nanoparticle. Associated references are 1. Holley et al. (1965), 2. Kim et al. (1973), 3. Berget et al. (1977) and Chow et al. (1977a), 4. Peattie (1979) and Peattie and Gilbert (1980), 5. Kruger et al. (1982) and Guerrier-Takada et al. (1983), 6. Greider and Blackburn (1985), 7. Altuvia et al. (1989), 8. Ellington and Szostak (1990) and Robertson and Joyce (1990) and Tuerk and Gold (1990), 9. Grundy and Henkin (1993), 10. Daros and Flores (1995), 11. Shen et al. (1999), 12. Ban et al. (2000), Wimberly et al. (2000), Harms et al. (2001), and Yusupov et al. (2001), 13. Nocker et al. (2001), 14. Mironov et al. (2002) and Winkler et al. (2002), 15. Wachter et al. (2007), 16. Lee et al. (2009), 17. Seidelt et al. (2009) and Armache et al. (2010), 18. Filichkin et al. (2010), 19. Jinek et al. (2012), 20. Ding et al. (2014) and Rouskin et al. (2014), 21. Bieri et al. (2017) and Perez Boerema et al. (2018), 22. Alves et al. (2017) and Cognat et al. (2017), 23. Su et al. (2018) and Chung et al. (2020), 24. Fajkus et al. (2019) and Song et al. (2019), 25. Dolgin (2021), 26. Waltz et al. (2020), 27. Ferrero-Serrano et al. (2022).

The sections of this review are organized to introduce each RNA class with a brief historical context regarding structural aspects (Fig. 1), followed by a discussion of features relevant or specific to plant biology. We discuss structure–function relationships for tRNAs, telomerase, the ribosome, the spliceosome, ribozymes, RNA thermometers, riboswitches, G-quadruplex (GQ)-forming RNAs, long noncoding RNAs (lncRNAs), and the structural diversity encompassed by the entire transcriptome, i.e. the “structurome.” (We have omitted the discussion of small RNA structure–function relationships in plants because this topic is covered by other articles in this Focus Issue.) We conclude by discussing unknowns and exciting prospects in the RNA structure field.

## Rock-like RNAs: tRNA, telomerase RNA, the ribosome, and the spliceosome

### tRNAs and their related sequences

**Figure 2.**
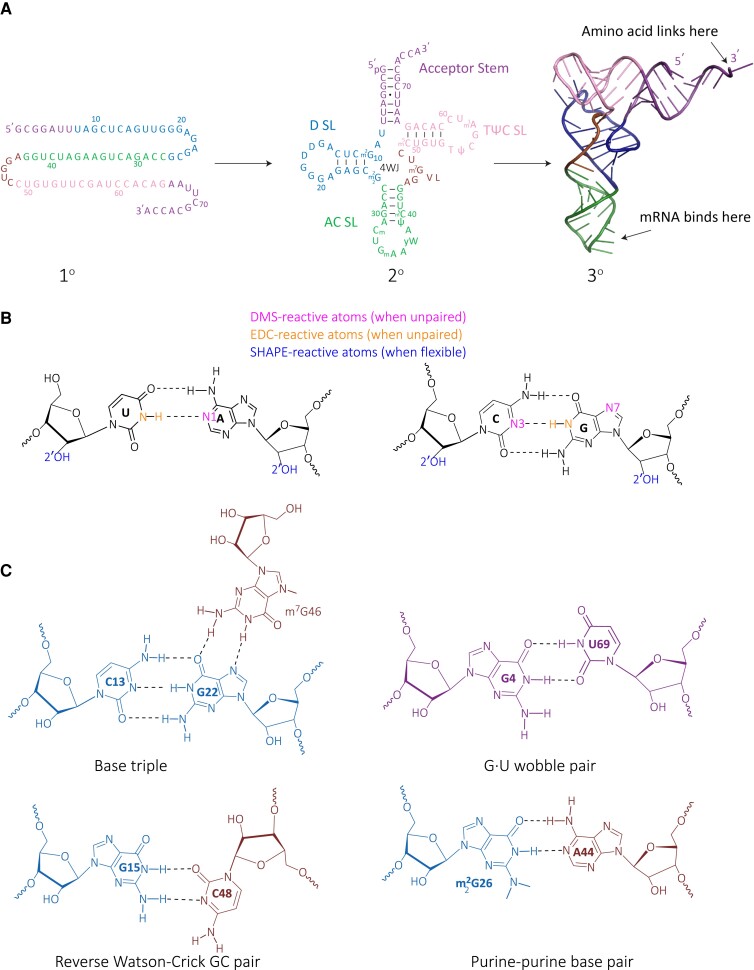
Features of tRNAs. A) Hierarchical folding of tRNA. Shown are the primary (1°), secondary (2°), and tertiary structures (3°) of tRNA, with the various SLs color coded. In the secondary structure, the D SL, anticodon SL, variable loop, TψC SL, and the acceptor stem are noted, as is the 4-way junction (4WJ). In the tertiary structure, the point of attachment of the amino acid and the site of binding of the mRNA codon are noted. B) Watson–Crick base pairing. Sites of modification by structure probing DMS, EDC, and SHAPE reagents are indicated. C) Non-Watson–Crick base pairing with coloring corresponding to the tRNA in A, illustrating a base triple, reverse Watson–Crick GC pair, G•U wobble pair, and purine–purine base pair. All images are from PDB ID: 4TNA for yeast tRNA^Phe^. Validation reports are available for this structure at the Protein Data Bank: https://www.rcsb.org/.

tRNA was first identified in the 1960s (Fig. 1) as an ∼75 nt RNA that canonically serves as an adapter between mRNAs and the ribosome. It was so named because it *transfers* information from mRNA to the growing polypeptide chain. The sequence and secondary structure of tRNA were worked out in 1965 by Robert Holley (Holley et al. 1965) who predicted several conformations (secondary structures) for tRNA including the native “cloverleaf” conformation (Fig. 2A). He also predicted rod-like conformations, which may, in fact, have biological relevance (see below). The cloverleaf secondary structure folds hierarchically (i.e. secondary structure before tertiary structure) into an “L-shaped” tertiary structure (Baldwin and Rose 1999; Tinoco and Bustamante 1999) (Fig. 2A). tRNAs are notable for being the most heavily modified RNAs, with an average of 13 modified nucleotides per 75 nt RNA (∼17%). Over 80 different types of posttranscriptional covalent modifications to tRNA are known (Tuorto and Lyko 2016).

tRNA contains a treasure trove of RNA secondary and tertiary structural motifs. The secondary structure of tRNA is marked by several key features. Three of these are hairpins or SLs, each comprised of a local fold of a short 4–5 canonical base pair (Fig. 2B) stem and a large 7–8 nucleotide loop. These SLs are named for their loop characteristics: the “D loop” because it has several dihydrouridine modifications, the “anticodon loop” because it contains the 3-nucleotide anticodon that base pairs with the codon in the mRNA, and the “TψC-loop” because it contains the characteristic TψC sequence. Nestled between the anticodon stem and the TψC stem is the variable loop, aptly named because it varies in size. Another feature is the acceptor stem, a long-range 7 base pair helix between the first and last parts of the tRNA. The acceptor stem and D-, anticodon, and TψC-SLs form a 4-way junction (4WJ) giving rise to the cloverleaf secondary structure. There is also the characteristic 3-nucleotide single-stranded CCA at the 3′-end that attaches to the amino acid through an aminoacyl bond.

The tertiary structure of tRNA (Fig. 2A) is marked by the acceptor stem at the short end of the L-shape and the anticodon at the long end of the L-shape. The L-shape fits perfectly into the ribosome and allows the anticodon to base pair with the mRNA and the acceptor stem to donate an amino acid to the polypeptide chain. The L-shape is formed by a number of long-range tertiary interactions most notably between the D and the TψC loops, as well as involving the variable loop. Space limitations prevent full exposition of the many tertiary interactions in tRNA but it is noteworthy that these involve diverse non-Watson–Crick interactions such as base triples, reverse Watson–Crick GC pairs, G•U wobbles, and purine–purine base pairs (Fig. 2C). These unusual pairings, which lock in the tRNA structure and make it “rock-like,” can be accommodated because the helices are short and the loops are large, leading to imperfections that accommodate otherwise unwieldy pairings. tRNA was the first functional RNA to have its structure worked out, with crystal structures solved in the 1970s (Kim et al. 1973, 1974) (Fig. 1), and it stands as one of the most fascinating of all structures in nature, linking chemistry and biology. We have recently advanced a method called “tRNA Structure-seq” for probing the structure of tRNAs in vivo (Yamagami et al. 2022). This method, which revealed tRNA misfolding during heat stress in *Escherichia coli*, may prove helpful in elucidating further tRNA biology, including in plants.

The biogenesis of tRNA is complex and involves processing of precursor tRNAs, which are transcribed in the nucleus by RNA Pol III (Phizicky and Hopper 2015). The tRNA is cleaved at the 5′-end by the endonuclease RNase P and at the 3′-end by RNase Z, followed by the addition of the 3′-CCA tail to produce mature tRNAs, which are exported to the cytoplasm. Once in the cytoplasm, short anticodon-proximal introns, present in a subset of tRNAs, are removed by a set of 3 enzymes (Phizicky and Hopper 2015). The now mature tRNAs can participate in their familiar roles in translation, but some undergo processing to generate tRNA-derived fragments (tRFs) with novel functions. There are several classes of tRFs: those containing the 5′-end including tRF-5a, tRF-5b, and 5′-tRNA half, which are approximately 18, 24, and 34 nt long, respectively, and those containing the 3′-end including tRF-3a, tRF-3b, and 3′-tRNA half, which are 18, 22, and 31–40 nt long, respectively. The main processing enzyme in the biogenesis of tRFs (Su et al. 2020) appears to be the nonspecific single-strand nuclease RNase T2 (Megel et al. 2019; Su et al. 2020) but the details remain to be elucidated. In metazoans, tRFs often come from hypomodified tRNAs. For example, in Drosophila, 5-methylation of C38 inhibits tRNA cleavage, while in humans Q and 2′-O-methylation of C34 in the anticodon loop each inhibit cleavage. One possibility is that modifications help fold the tRNA, protecting it from the single-strand RNase T2, whereas loss of modification or presence of stress unfolds or misfolds the tRNA making it susceptible to cleavage.

In plants, tRNA of course performs its expected role as an informational molecule in translation. An additional important consequence of tRNA processing in plants is the production of tRFs, which do not fold like tRNA but act as small RNAs. Another key role of tRNA is the formation of tRNA-like structures (TLSs), which can fold into a cloverleaf structure. As a point of nomenclature, we retain the conventions in the field of “tRFs” and “TLSs,” even though “tRNA” is represented as “tR” in the acronym “tRFs” and as “T” in “TLSs.” In this section, we highlight some of the key recent advances regarding the roles of plant tRFs and TLSs in RNA trafficking, translation, gene silencing, and abiotic stress response (Ma et al. 2021b).

Plant tRFs are generated in response to numerous abiotic stresses, including phosphate starvation, temperature, oxidative stress, and UV light (Ma et al. 2021b), although in many cases functional roles remain to be elucidated. Further study of the structures of tRNA and its derivatives in vivo and during stress may help connect the biogenesis of tRFs to tRNA structure and modification. It is not impossible that misfolding of tRNAs into rod-like structures, first predicted by Holley et al. (1965) and observed in our tRNA Structure-seq data (Yamagami et al. 2022), could be part of the pathway.

Plant tRFs have been suggested to interfere with ribosome activity and thus inhibit translation, albeit only a few studies have addressed this topic to date (Zhang et al. 2009; Lalande et al. 2020). This view was first proposed by Zhang et al. (2009), who demonstrated that RNAs isolated from the phloem sap of pumpkin (*Cucurbita maxima*) inhibited in vitro translation in a wheat germ extract system. Interestingly, this inhibition was lost if the RNA was extracted using a denaturing protocol, suggesting an essential role of RNA structure. Although phloem sap contains a diversity of RNA species, a specific role for tRFs was demonstrated in targeted experiments wherein tRFs generated from yeast tRNA, but not intact tRNA or nucleotides, were shown to inhibit the in vitro translation of *FLOWERING LOCUS T*, *luciferase*, and *AtMPB2C*. tRFs’ function in translation suppression was further validated by a mimic study using a brome mosaic virus RNA in vitro translation system with yeast artificial tRNA fragments (Zhang et al. 2009). Recently, Arabidopsis tRFs deriving from tRNA^Ala^(AGC) and tRNA^Asn^(GUU), but not tRFs known to be associated with AGO complexes (see below), inhibited green fluorescent protein synthesis in a wheat germ extract system, and the G18 and G19 residues of the D loop were found to be essential for this repression (Lalande et al. 2020). Interestingly, this report indicated that the Arabidopsis tRFs were enriched in the ribosomal fractions, suggesting that tRFs are capable of binding and interacting with polysomes, thereby globally inhibiting translation in plants.

tRFs were found to be synthesized in pollen via a microRNA-like Dicer-dependent machinery, as both miRNAs and 19 nt 5′-region tRFs (tRF-5s, see above), derived from mature tRNAs of the majority of codon types, were reduced significantly in *dcl1* (*Dicer-like 1*) T-DNA mutants (Martinez et al. 2017). In this report, libraries produced from AGO1-immunoprecipitated sRNAs contained abundant tRF-5s, indicating that they were incorporated into the AGO1 silencing complex. Resultant tRFs-AGO1 complexes cleave transposable element (TE) mRNAs thus protecting the pollen genome from TE activation.

**Figure 3.**
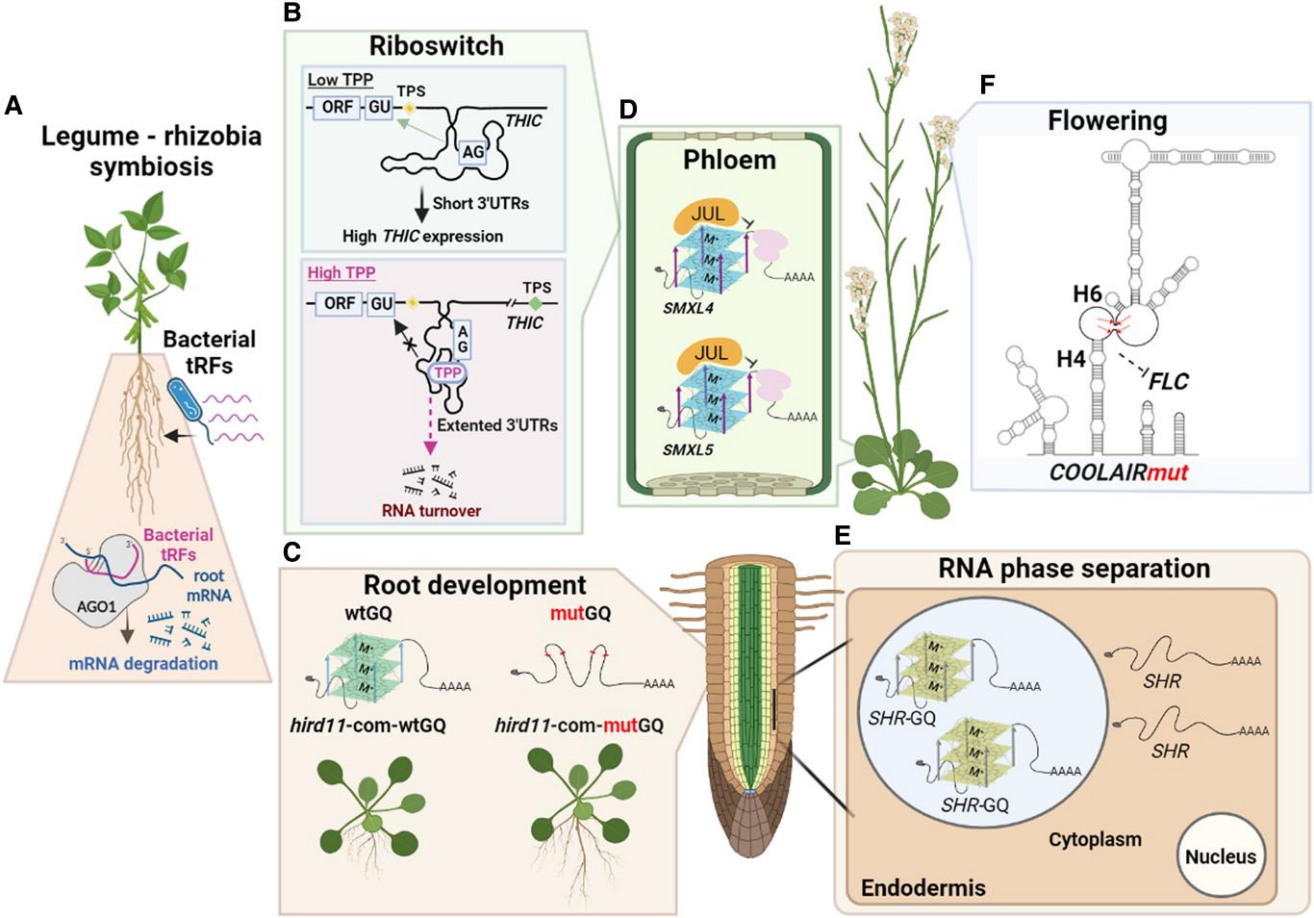
Schematic of RNA structure–function relationships in plant growth and development. A) tRFs play important roles in the symbiosis between legumes and rhizobia bacteria. tRFs produced by bacteria (*B. japonicum*) are delivered into AGO1 (Argonaute) complexes of soybean root cells, resulting in the degradation of nodule-suppressing plant mRNAs and thus promoting nodule initiation and development. B) TPP-sensing riboswitches regulate *THIC* mRNA accumulation through the control of pre-mRNA splicing and 3′ end processing (Bocobza et al. 2007; Wachter et al. 2007). Upper: when TPP concentrations are low, the aptamer interacts with a 5′-splice site GU within the 3′-UTR and prevents splicing, with consequent retention of a poly-A cleavage site (transcription processing site or TPS, upstream/yellow) within a 3′-UTR intron that causes 3′UTRs cleavage and polyadenylation for high expression of *THIC*. Lower: in the presence of elevated TPP concentrations, the aptamer sequence no longer base pairs with the 5′-splice site, making it accessible for splicing, and consequently removing the TPS and extending the length of the 3′-UTR at the 3′-splice site AG. This 3′UTR is long (as noted by the break in the sequence) and has a different TPS (downstream/green), which leads to increased RNA turnover and thus reduced expression of *THIC.* C) An example of GQ structure and its function in root development in *HIRD11* mRNA (Yang et al. 2020b). In a *hird11* mutant complementation study, mutGQ-complemented transgenic plants (lacking *HIRD11* GQ structure) showed longer roots than those of wtGQ-complemented plants. D) In the phloem, transcription factor JUL and the promoter of *SMXL4/5* genes are key regulators of phloem differentiation. JUL directly binds and induces a GQ within the 5′ UTR of *SMXL4/5* mRNAs; this JUL-induced GQ-*SMXL4/5* complex suppresses *SMXL4/5* translation and consequently inhibits phloem differentiation (Cho et al. 2018). E) In the endodermis, *SHR* mRNA contains a GQ structure that can elicit liquid–liquid phase separation in vitro (Zhang et al. 2019). This RNA structure-initiated phase separation is suggestive of an important biological function in the translational regulation of *SHR*, which is a major player in root development. F) In the flowering process, the depicted mutant version (red arrows indicate the mutation sites) of the *COOLAIR* natural antisense lncRNA of *FLC* suppresses *FLC* transcript abundance and accelerates flowering (Hawkes et al. 2016; Yang et al. 2022a).

Gene regulation by tRFs also can occur between kingdoms (Fig. 3A). In the symbiosis between legumes and Rhizobia bacteria, the bacteria enter the plant and stimulate the formation of nodules, wherein the bacteria supply the plant with fixed nitrogen and the plant supplies the bacteria with carbohydrates. In soybean and its rhizobial partner, *Bradyrhizobium japonicum*, tRFs produced by the bacteria enter root cells via an unknown mechanism, where they are loaded into the AGO1 (Argonaute) complex and target sequence-homologous host genes for suppression, thereby promoting nodule initiation and development (Ren et al. 2019).

Small interfering RNAs (siRNAs), microRNAs (miRNAs), rRNAs, mRNAs, and tRNAs also can move locally in plants via plasmodesmata from cell to cell and/or over long distances by entering the phloem vascular system (Thieme et al. 2015; Kehr and Kragler 2018; Tian et al. 2020). In 2015, Christoph Thieme and co-workers conducted the first genome-wide study of RNA trafficking, using Arabidopsis heterografted plants with different Arabidopsis accessions (ecotypes) as rootstock and scion to allow assignation of the mRNAs to their source based on allelic differences between these genotypes. Their study revealed that over 2,000 endogenous mRNAs were transported, both from roots to shoots and from shoots to roots, via the phloem vascular system (Thieme et al. 2015). Such long-distance movements may be facilitated by TLSs. In 2016, TLSs were shown to facilitate mRNA movement by analysis of wild-type phloem sap across chimeric graft junctions (transgenic tissue: wild-type tissue). In particular, GUS reporter mRNAs were efficiently transported to the scion phloem upon addition to their 3′-UTR of a full-length tRNA^Met^ or tRNA^Gly^ (but, interestingly not a tRNA^Ile^) or a stem-bulge-SL-forming tRNA partial sequence (Zhang et al. 2016). In particular, the anticodon and T-loops were shown by these authors to be most important for transport. The authors ascertained that ∼11% of Arabidopsis transcripts have TLSs in their CDS or 3′-UTR, so this may be a quite general endogenous mechanism; these are enriched in genes encoding mobile transcripts and are typically close (∼100–200 bp) to the end of the gene. In addition, dicistronic mRNA-tRNA transcripts were also found to be over-represented in mobile RNAs (Wang et al. 2019b), again suggesting that structures found in tRNAs promote mobility, although the underlying mechanism, and the reasons for the efficacy of some tRNA sequences and not others, remains unknown. In further support of the role of TLSs and tRNAs in long-distance mRNA transport, recently, 2 long-distance mobile mRNAs, *CHLOROPHYLL A/B BINDING PROTEIN 1* (*CAB1*, AT1G29930.1) and *LIGHT-HARVESTING COMPLEX GENE 3* (*Lhca3*, AT1G61520.1), were investigated and both RNAs possess conserved 3WJ and MWJs (multi-way junctions) in cloverleaf-like structures as revealed by RNA structural profiling using dimethyl sulfate (DMS)-MaPseq (Wang et al. 2019b) (Table 1), a technique that is explained later on in the methods section.

**Table 1.** Methods for genome-wide high-throughput analysis of RNA structures by chemical probing strategies

Approach	Full name	Chemical probe	Species; in vitro or in vivo	Comments, First report
CIRS-seq (RT stops)^a^	Chemical inference of RNA structures sequencing	DMS, CMCT	• E14 ESCs (*M. musculus*); in vitro (Incarnato et al. 2014)	Genome-wide RNA structure sequencing with chemical probing on 4 nucleotides by 2 chemicals simultaneously in vitro (i.e. DMS on As, Cs, and CMCT on Gs, Us) (Incarnato et al. 2014)
Structure-seq (RT stops)	RNA structure sequencing	DMS	• *A. thaliana*; in vivo (Ding et al. 2014; Ding et al. 2015; Tack et al. 2020; Tang et al. 2016) • *O. sativa*; in vivo (Deng et al. 2018)	In vivo genome-wide RNA structure sequencing (Ding et al. 2014)
DMS-seq (RT stops)	Dimethyl sulfate-modified RNA sequencing	DMS	• *S. cerevisiae BY4741,* K-562 cells (*H. sapiens*); in vitro and in vivo (Rouskin et al. 2014) • *E. coli*; in vitro and in vivo (Zhang et al. 2018)	In vivo genome-wide RNA structure sequencing (Rouskin et al. 2014)
SHAPE-MaP (MaP)^b^	Selective 2′-hydroxyl acylation analyzed by primer extension (SHAPE) mutational profiling	SHAPE (NAI, 1M7, 2A3)	• *HIV-1;* in vitro (1M7, (Siegfried et al. 2014) • *SARS-CoV-2;* in vivo and in vitro (NAI; (Manfredonia et al. 2020) • *E. coli*, *B. subtilis*, HEK293 cells (*H. sapiens*); in vivo and in vitro (2A3; (Marinus et al. 2021) • *T. turgidum;* in vivo (NAI; Yang et al. 2021)	In vitro RNA structure sequencing probing of 4 nucleotides at the sugar by mutational profiling (Siegfried et al. 2014)
icSHAPE (RT stops)	In vivo click selective 2′-hydroxyl acylation and profiling experiment	SHAPE (NAI-N3)	• mESCs (*M. musculus*); in vitro and in vivo (Spitale et al. 2015) • *SARS-CoV-2*; in vitro and in vivo (Sun et al. 2021)	In vivo RNA structure sequencing coupled with copper-free click chemistry of adding biotin specifically to NAI-N3-modified RNAs (Spitale et al. 2015)
rG4-seq (RT stops)	RNA GQ sequencing	Pyrdiostatin (PDS)	• HeLa cells (*H. sapiens*); in vitro (Kwok et al. 2016; Yeung et al. 2019) • *E. coli, P. aeruginosa*; in vitro (Shao et al. 2020) • *P. falciparum*; in vitro (Dumetz et al. 2021)	Transcriptome-wide RNA GQ (rG4) profiling coupled with rG4-specific PDS ligand (Kwok et al. 2016)
Structure-seq2 (RT stops)	RNA structure sequencing 2	DMS	• *O. sativa*; in vivo (Ritchey et al. 2017; Su et al. 2018) • *B. subtilis*; in vivo (Ritchey et al. 2020)	In vivo data with improved read coverage and reduced ligation bias when compared with Sructure-seq (Ritchey et al. 2017)
DMS-MaPseq (MaP)	Dimethyl sulfate mutational profiling with sequencing	DMS	• *S. cerevisiae* R64, HEK293 cells (*H. sapiens*), and *D. melanogaster*; in vivo (Zubradt et al. 2017) • *A. thaliana*; in vivo (Wang et al. 2019b) • *A. thaliana*; in vivo and in vitro (Song et al. 2019) • *SARS-CoV-2*; in vivo and in vitro (Manfredonia et al. 2020) • *HIV-1*; in vivo and in vitro (Tomezsko et al. 2020) • *SARS-CoV-2*; in vivo (Lan et al. 2022)	In vivo DMS RNA structure probing by mutational profiling. (Zubradt et al. 2017)
CAP-STRUCTURE-seq (RT stops)	5′CAP-enriched and 3′ poly(A)-enriched RNA structure sequencing	SHAPE (NAI)	• *A. thaliana*; in vivo (Yang et al. 2020a)	RNA SHAPE structure sequencing of 5′cap-enriched and 3′ poly(A)-enriched intact mRNAs (Yang et al. 2020a)
SHALiPE-Seq (RT stops)	Selective 2′-hydroxyl acylation with lithium ion-based primer extension	SHAPE (NAI)	• *O. sativa*, *A. thaliana*; in vitro and in vivo (Yang et al. 2020b)	Genome-wide rG4 mapping by SHAPE with lithium ion-based primer extension (Yang et al. 2020b)
icSHAPE-MaP^1^ and RIP-icSHAPE-MaP^2^ (MaP)	^1^In vivo click selective 2′-hydroxyl acylation by primer extension and mutational profiling ^2^icSHAPE-MaP with RNA immunoprecipitation	SHAPE (NAI-N3)	• HEK293 cells (*H. sapiens*); in vitro and in vivo (Luo et al. 2021)	In vivo genome-wide icSHAPE-MaP method on sRNA (Luo et al. 2021)
tRNA structure-seq (MaP)	tRNA structure sequencing	DMS	• *E. coli* BW25113; in vitro and in vivo (Yamagami et al. 2022)	Genome-wide mutational profiling of tRNA structurome coupled with DMS (Yamagami et al. 2022)

“RT stops” indicates that the chemical probe modification is detected by the RT stop that it causes.

“MaP” indicates that the chemical probe modification is detected by its induction of a mutation in the cDNA following RT.

### Telomerase RNA

**Figure 4.**
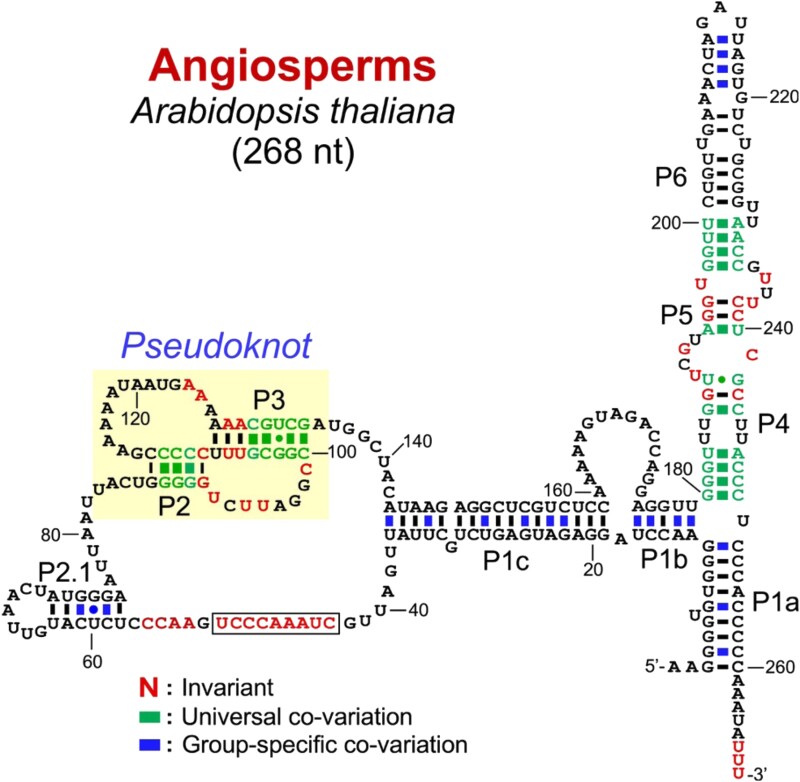
Telomerase RNA from Arabidopsis. The 268 nt RNA telomerase RNA from *A. thaliana*, referred to as AtTR, with the conserved pseudoknot domain highlighted in yellow and the 9 nt 5′-CUAAACCCU template boxed. This region, known as the T-PK domain, is closed by the TBE which is the extensive helix spanning nts 36 to 143. Reprinted from Song et al. (2019), Fig. 3. Copyright 2019 National Academy of Sciences.

The ends of chromosomes, known as telomeres, have G-rich repeats that are added by an RNA–protein complex (RNP) known as telomerase, which comprises telomerase RNA (TR) and telomerase reverse transcriptase (TERT). The C-rich template for the repeats is contained within TR, which folds into a complex structure involving a conserved pseudoknot (Fig. 4). Other structural features in TR facilitate DNA binding and enzymatic regulation.

The first publication on the discovery of telomerase was in 1985 (Fig. 1) (Greider and Blackburn 1985) and subsequent studies uncovered conserved and divergent elements in TRs (Song et al. 2019). The most conserved RNA domains function in catalysis (Qi et al. 2013) and are contained within the template-pseudoknot (T-PK) domain, which harbors 1.5–2 copies of the complement to the DNA telomeric repeat near the 5′-end of the T-PK and the TERT-interacting PK near the 3′-end of the T-PK (Fig. 4). The entire T-PK domain is bounded by an extensive stem known as the template boundary element (TBE) (Chen and Greider 2003).

Telomerase RNA in plants has recently been elucidated. The TR from Arabidopsis has been identified as a conserved and essential 268 nt PolIII transcript termed AtTR (Figs. 1 and 4) (Fajkus et al. 2019). Fajkus et al. (2019) found and experimentally confirmed in vitro and in vivo that the Arabidopsis telomere 7 nt repeat of (TTTAGGG)*_n_* is templated by AtTR's 9 nt stretch of 5′-CUAAACCCU. They also identified TRs in diverse land plants, including crop and model plants. Dorothy Shippen and colleagues (Song et al. 2019) used next-generation sequencing analysis of TERT-associated RNAs to independently identify AtTR as the functional TR in Arabidopsis, which they also experimentally confirmed in vitro and in vivo. They provided a structural model for TR built from phylogenetic analysis of divergent plant lineages (Fig. 4), which was supported by chemical probing of SHAPE (selective 2′-hydroxyl acylation and primer extension) reactivity in vitro and DMS-MaPseq in vivo (see Table 1 and “Paper-like RNAs II: Many Conformations” for descriptions of these techniques). Recent analysis of TRs from the Fajkus group (Fajkus et al. 2021) concludes that the T-PK and TBE are highly conserved and describes the evolution of the earliest eukaryotic TRs.

### Ribosomes

**Figure 5.**
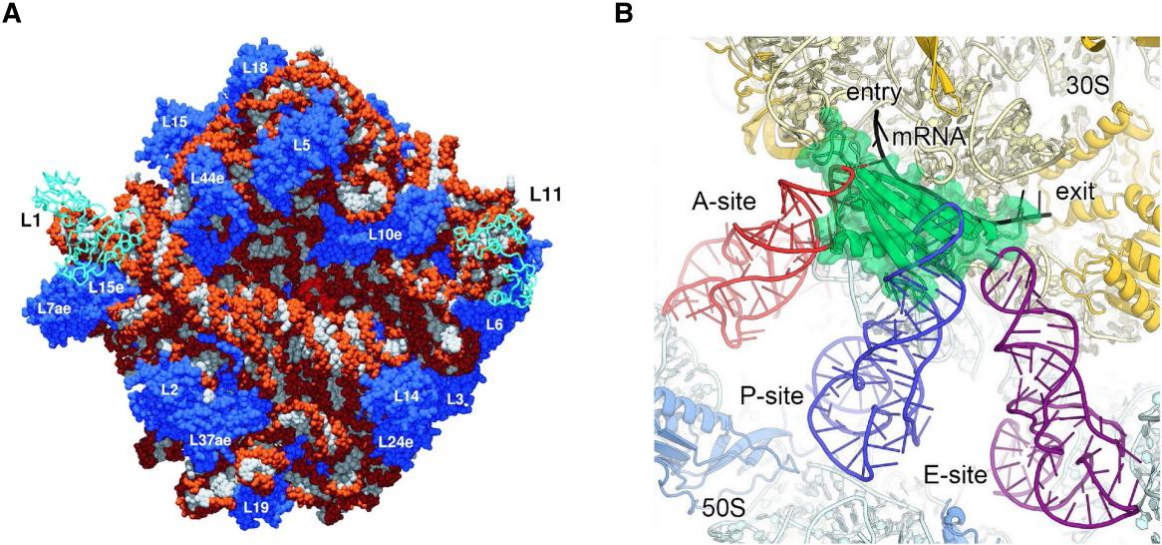
Ribosome structures. A) Structure of the 5S and 23S rRNA from the halophilic archaeon *Haloarcula marismortui.* The bases are white and the sugar-phosphate backbones are orange. The CCA acceptor stem model of the reaction intermediate for peptide bond formation is red and the numbered proteins are blue. The L1 and L11 proteins positioned at lower resolution are in cyan, where “L” is for LSU that comprises 5S and 23S rRNA. From PDB ID: 1FFZ (Nissen et al. 2000). Reprinted with permission from AAAS. B) Translation inhibition by the translation factor pY. The chloroplast 70S (yellow and blue):pY (green) complex is superposed with the bacterial A-, P- and E-site tRNAs (red, blue, and purple structures, respectively) including mRNA from the crystal structure of the *Thermus thermophilus* 70S ribosome. Note how pY is bound in the mRNA (black) channel and prevents tRNA binding. From superposition of PDB ID: 5MMI (50S) and 5MMJ (30S), with tRNAs and mRNAs from 4V51 (Bieri et al. 2017). Reprinted with permission from Wiley. Validation reports are available for these structures at the Protein Data Bank: https://www.rcsb.org/.

The ribosome is an extraordinarily complex molecular machine comprised of RNA and proteins that synthesizes proteins in the process of translation. It is massive at ∼2.5 MDa in bacteria and up to 4.6 MDa in eukaryotes, with approximately two-thirds of the mass coming from RNA (Yusupova and Yusupov 2015). Ribosomal RNAs have multiple covalent modifications, and there is growing evidence that ribosomes can be specialized via specific ribosomal proteins for additional functions such as translation of specific mRNAs (Shi et al. 2017). The basic composition and assembly pathways of ribosomes have been studied for many years (Nomura 1973; Williamson 2003; Woodson 2011), but structures of the ribosome were elucidated only in the last few decades. Seminal ribosomal structural studies at near-atomic resolution by Thomas Steitz, Venkatraman Ramakrishnan, Ada Yonath, Harry Noller, and colleagues (Fig. 1) were performed by heroic X-ray crystallographic efforts (Ban et al. 2000; Wimberly et al. 2000; Harms et al. 2001; Yusupov et al. 2001; Ramakrishnan 2002). Ribosomes consist of the small subunit (SSU) and large subunit (LSU), which cooperate via a complex network of interactions. Ribosomes generally reside in the cytoplasm and in mitochondria and plastids. Structures of the organelle-specific ribosomes are being unveiled and are revealing novel features (see below). High-resolution structures of the cytosolic ribosome have uncovered how tRNAs move through the A, P, and E sites, as well as the pathway for mRNA entry and exit. One of the most striking discoveries about the ribosome is that it is a ribozyme: proteins are present throughout the ribosome but are absent near the active site, marked by the transition state analog (Nissen et al. 2000) shown in Fig. 5A, which captures the monolithic nature of the ribosome. Although the ribosome does exhibit some local dynamics (Nissen et al. 2000; Sanbonmatsu et al. 2012), the extensive nature of base pairing, tertiary structure, and protein interactions, much of which is structurally conserved across biological species, help lock in the structure of the ribosome and thus make it “rock-like.”

Space limitations prevent a fuller treatment, but ribosomal structural biology remains an active and vibrant field. Cryo-EM now allows near-atomic resolution of large RNA–protein structures to be solved without the need for crystallization, even on relatively low abundance materials with some level of conformational and compositional heterogeneity. Indeed, the cryo-EM revolution has enabled near-atomic structures of RNPs such as the ribosome and spliceosome (see below) (Nogales and Scheres 2015; Fica and Nagai 2017; von Loeffelholz et al. 2017) and increasingly of RNA-only macromolecules (Kappel et al. 2020). Moreover, structures of the ribosome are now solved faster and at near-atomic resolution by cryo-EM to reveal novel features including covalent modifications of the RNA and protein, as evidenced by a recent structure of the 70S ribosome from bacteria at 2.0 Å resolution (Watson et al. 2020). These key technical advances open the door to insights into how specialized ribosomes and RNA modifications help regulate translation.

Cytosolic, chloroplastic, and mitochondrial ribosomes in plants have both canonical and specialized aspects. For example, recent work suggests that the plant cytoplasmic ribosome can function as a metabolite sensor. This function was first established in bacteria (Wilson et al. 2016) for sensing of tryptophan (Bischoff et al. 2014) and the antibiotic erythromycin (Arenz et al. 2016), and extends to plants where sensing has been established for diverse metabolites including sucrose, spermidine, spermine, thermospermine, galactinol, phosphocholine, and ascorbate (van der Horst et al. 2020). This plant ribosome-specific function is novel on several levels, not the least of which is that there are no known riboswitches for these metabolites, perhaps making the ribosome the largest riboswitch ever. Smeeken and colleagues (van der Horst et al. 2020) presented evidence that sucrose can bind in the exit tunnel along with a nascently translated conserved peptide from the upstream open reading frame (CPuORF) to block translation. Given that hundreds of plant genes encode CPuORFs, this novel mechanism could play a central role in metabolite sensing and the control of translation. In addition, Joachim Kopka and co-workers point out that each ribosomal protein in Arabidopsis has multiple paralogs, leading to the potential for tremendous combinatorial diversity of the ribosome and, therefore, for functional diversity (Martinez-Seidel et al. 2020). Specialized ribosomes are an emerging area of ribosomal research that could unveil new levels of control of gene expression in plants.

There are 2 plant-specific structures of the ribosome, 1 in chloroplasts and 1 in mitochondria. First, we consider the chloroplast ribosome structures, focusing on the pY translation factor complex from spinach leaves, which was solved by cryoEM to near-atomic resolution at ≥3.4 Å (Fig. 5B) (Bieri et al. 2017; Perez Boerema et al. 2018). Chloroplasts arose by endosymbiosis and so contain their own genome, and their ribosomes are similar to those of bacteria. The chloroplast ribosome structure is unusual in that there is a 4.5S rRNA, thought to have been derived from 23S rRNA, as well as 5 plastid-specific proteins. These proteins interact with rRNA and stabilize chloroplast-only RNA segments. More subtly but equally specialized are extensions of the known ribosomal proteins that remodel all of the entries and exits in the ribosome, including the mRNA entry and exit tunnels, the polypeptide exit tunnel, the signal-recognition particle (SRP) binding site, and the LSU binding site. These observations suggest specialization of the chloroplast ribosome in both its protein and RNA components. Revealed in exceptional clarity in Fig. 5B is the positioning of the plastid translation factor pY, which is involved in attenuating translation in response to darkness and cold (Bieri et al. 2017). The ribosome-pY co-structure reveals pY interacting with 16S rRNA nucleotides at the A- and P-sites, where it inhibits translation by competing with tRNAs for binding (Fig. 5B). This superposition figure illustrates this clearly, in which pY binds to 16S rRNA in a way that blocks tRNAs from their A- and P-sites.

Mitochondria were also acquired by endosymbiosis and contain specialized ribosomes referred to as “mitoribosomes.” The overall process appears to involve duplication of mitochondrial genes of bacterial origin, expansion of protein size, and recruitment from the host of originally non-ribosomal mitochondrial proteins such as proteins originally functioning as tRNA synthetases (O’Brien 2002; Smits et al. 2007; Gray 2012). Plant mitoribosomes have expanded rRNA segments and over 10 plant-specific ribosomal proteins that are mostly pentatricopeptide repeat proteins (PPR). The evolutionary route taken by plants has been referred to as a “constructive phase” of ribosome evolution since rRNA was extended and proteins were added (Waltz et al. 2020). Distinct from mammals and *Trypanosoma*, wherein mitoribosomes obtained more novel ribosomal proteins and drastically decreased rRNA size, plant mitoribosomes lost few proteins and instead gained extended rRNA that binds novel ribosomal proteins. The expansions of the 5S, 18S, and 26S rRNAs make the plant mitochondria SSU and LSU rRNAs significantly larger than prokaryotic rRNAs—20% and 9% larger, respectively. Yaser Hashem and co-workers solved a 78S mitoribosome from cauliflower at near-atomic resolution (Waltz et al. 2020). Their main finding is that ribosomal PPR proteins interact with the mitoribosome-specific rRNA expansions. This structure continues the theme of organelle-specific proteins interacting with organelle-specific RNA species. Finally, it is notable that green algae such as Chlamydomonas have a fragmented mitoribosome comprised of 13 rRNA fragments. Cryo-EM studies reveal how these fragments assemble to make a stable mitoribosome by base pairing with each other in the SSU and by charge interactions with novel ribosomal proteins in the LSU (Waltz et al. 2021). It is clear that plants have evolved elegant strategies to elaborate the ribosome for organelle-specific function.

### Spliceosome

**Figure 6.**
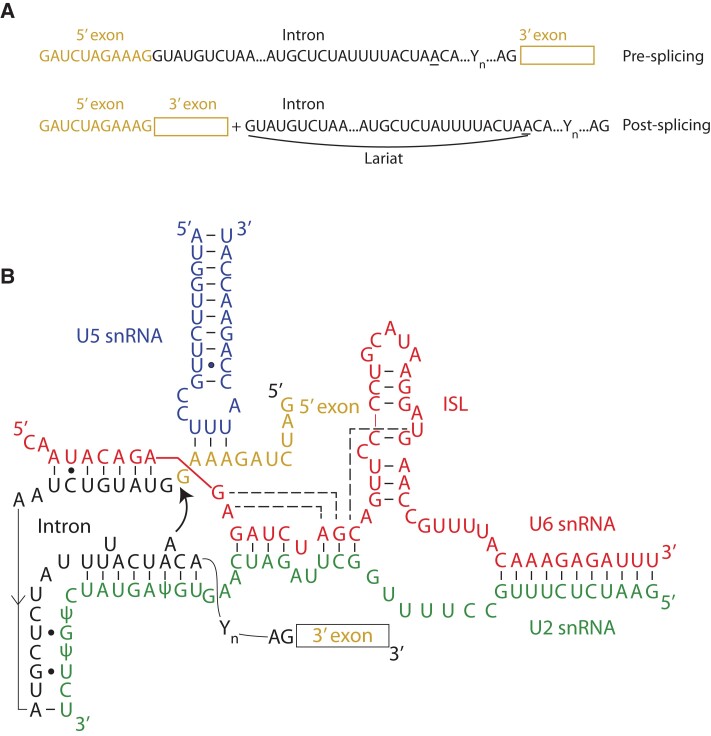
Spliceosome base pairing and reaction. A) Sequence of an mRNA pre-splicing and post-splicing with the intron lariat depicted. The underlined “A” is the branch point adenosine, “Yn” depicts the ∼15–20 nt polypyrimidine tract (where “Y” is the IUPAC abbreviation for pyrimidine), and the final “AG” is the conserved dinucleotide sequence immediately adjacent to the 3′-splice site. B) Spliceosome snRNA interactions before the first-step splicing reaction. This complex involves the 3 snRNAs U2 (green), U5 (blue), and U6 (red). There is extensive base pairing between U2 and U6, while U5 interacts only weakly with the 5′-exon (gold). The U6 snRNA also interacts with the intron (black) and forms an intramolecular SL (ISL). The AGC of U6 snRNA shows dashed lines that form the catalytic triad. The bulged A in the intron sequence UACUAAC is the branch point adenosine and uses its 2′hydroxyl as a nucleophile to attack the 5′-splice site as shown. Adapted with permission from Fica and Nagai (2017), Fig. 4. Copyright Springer Nature.

The discovery that genes are split into exons and introns (Fig. 6A) was made by Phillip Sharp, Richard Roberts, and co-workers in 1977 (Fig. 1), nearly 50 yr ago (Berget et al. 1977; Chow et al. 1977a, 1977b). The complex responsible for removing the introns and joining the exons is a remarkable RNA–protein machine referred to as the “spliceosome,” which is found in the nucleus of eukaryotic cells. As a massive RNP complex, we classify it as rock-like; however, given that it catalyzes bond cleavage, it could be considered scissors-like, while its ability to reassemble throughout the splicing cycle among 10 different conformations (Fica and Nagai 2017) also confers a paper-like attribute. The spliceosome is large and its architecture complex. It consists of 5 small nuclear RNAs (snRNAs) named “U1,” “U2,” “U4,” “U5,” and “U6,” which bind to specific proteins to form small nuclear ribonucleoproteins (snRNPs) bearing the same names. The snRNAs are ∼150 nt in length and often modified, such as several key pseudouridines (ψ) in U2 snRNA. The snRNPs interact with the precursor mRNA at the splice sites and an internal branch point adenosine to orchestrate the 2 steps of splicing (Fig. 6B). The 2 steps of nuclear pre-mRNA splicing are mechanistically identical to those of Group II intron, and the second step is also parallel to that of Group I intron (both of these introns are discussed further in the section on ribozymes).

Over the last 50 yr, many advances have been made in uncovering splicing mechanisms. For instance, it is known that the spliceosome assembles on the precursor mRNA in 2 steps that involve base pairing between some of the snRNAs, for instance, U4–U6. Subsequently, U6 and U2 base pair with each other and with the 5′-splice site and the branch point A to guide the first step of splicing (Fig. 6). In this step, the branch point A attacks the 5′-splice site to form a lariat with the 5′-end of the intron and release the 5′-exon. In the second step of splicing, the 3′-end of the 5′-exon attacks the 3′-splice site to form the mature exon–exon product and release the lariat-containing intron. The spliceosome is then recycled and can reassemble on another mRNA.

It is notable that splicing does not always occur in a canonical fashion of joining exons in consecutive order (Choquet et al. 2022). Moreover, variations on the theme, known as “alternative splicing,” often occur. In humans, ∼95% of genes are alternatively spliced, and in plants, ∼70% of genes are alternatively spliced (Chaudhary et al. 2019). A variety of alternative splicing events are known, with the 2 largest categories being exon skipping and intron retention. Alternative splicing diversifies the gene products and provides regulation.

As mentioned, the spliceosome is an RNP machine and so an obvious target for structural studies. Nonetheless, a high-resolution structure of the spliceosome was elucidated only recently. This is despite earlier determination of atomic-resolution structures of a plethora of functional RNAs including tRNAs, self-splicing group I introns, Group II introns, ribozymes, riboswitches, and the ribosome, as detailed elsewhere in this review. There are several reasons for this delay. First, unlike tRNA and the ribosome, the spliceosome is relatively low in abundance. Second, the spliceosome is not entirely globular, making crystallization challenging. Finally, as described above, its composition and conformation change throughout the process of assembly and splicing, confounding preparation of a pure, homogeneous complex for X-ray crystallography. It was the cryo-EM revolution that ultimately allowed the structure of the spliceosome to be solved for the first time.

Pioneering work by a number of labs, summarized in several reviews, has uncovered structures of the spliceosome at key junctures in splicing including activation, positioning, catalysis, and conformational change (Fica and Nagai 2017). A structure has been solved for nearly every step in the splicing cycle, and these structures reveal exquisite and specific RNA–RNA and RNA–protein interactions. This is remarkable given the size of the spliceosome at ∼1.5 MDa. A few salient features of these structures are worth noting. The first is that, despite the size of the spliceosome, the snRNAs and the mRNA are held together by relatively weak interactions. For instance, Fig. 6B shows that the U2 and U6 snRNAs have intra- and intermolecular base pairing segments that are as short as 2 or 3 base pairs, while the 5′-exon is paired to U5 snRNA by only 3 AU base pairs. The second feature is that the spliceosome is a ribozyme, as revealed by metal ion rescue studies and structural studies that show an active site nearly identical to that of the catalytic RNA-only Group II intron (Fica et al. 2013). Remarkably, both the ribosome and spliceosome, despite being RNP machines, use only RNA to conduct bond breaking and making, suggesting that they may be remnants of an RNA World. Anecdotally, efforts to show that the ribosome and spliceosome could conduct chemistry without any proteins (i.e. completely in vitro) have failed, emphasizing the intimate nature of RNA–protein interactions in these machines despite proteins not directly participating in catalysis (Noller et al. 1992).

There are clear differences in alternative splicing in humans when compared with plants (Chaudhary et al. 2019). For example, exon skipping is greatly reduced in plants, from ∼40% in humans to only ∼8% in plants. On the other hand, intron retention is increased in plants by an even greater margin, from ∼5% in humans to ∼60% in plants. The consequence of intron retention often is the degradation of the transcript because of the generation of premature stop codons that precipitate nonsense-mediated decay, although some transcripts produce truncated proteins.

Alternative splicing is modulated by abiotic stresses, including low and high temperatures, drought, salinity, nutrients, light, and the circadian clock (Reddy et al. 2013; Chaudhary et al. 2019; Punzo et al. 2020). Plant responses to pathogens, both PAMP-triggered and effector-triggered immunity, also involve major changes in alternative splicing (Rigo et al. 2019; Kufel et al. 2022). A recent publication from a multi-lab collaboration in which long-read PacBio sequencing was performed on RNA samples from different Arabidopsis organs as well as from Arabidopsis plants subjected to 5 different abiotic stresses and 4 plant pathogens provides an invaluable transcriptomic resource. These data were combined with short-read Illumina datasets to provide an unparalleled annotation of transcription start sites, transcript end sites, and splice junctions. The resultant *Arabidopsis thaliana* Reference Transcript Dataset 3 (AtRTD3) contains over 169,000 distinct transcripts representing 24,344 genes and should greatly facilitate analysis of stress- and tissue-specific splicing (Zhang et al. 2022).

Stress affects the ratio and timing of alternative splicing, although the mechanism remains largely unknown. Given the exquisite but somewhat fragile structure of the spliceosome in terms of snRNA pairing strength (see above), one possibility is that stress sometimes leads to melting of snRNA-containing base pairs (snRNA, snRNA-snRNA, and snRNA-mRNA) (Fig. 6B), which would prevent the spliceosome from functioning, especially on mRNAs where the pairing of U5 or U6 snRNAs at the 5′-splice site is weak, or where the pairing of U2 snRNA to the branch point A is weak. It is also notable that plant introns tend to be AU-rich, suggesting weak base pairing (McCullough and Schuler 1997; Kufel et al. 2022). This would be congruent with the observation that increasing temperature leads to gain in alternative splicing of heat stress response genes (John et al. 2021).

Regarding stress-responsive alternative splicing in plants, the Arabidopsis *LETHAL UNLESS CBC7* (*LUC7*) protein was identified from RNA immunoprecipitation experiments and transcriptome analyses as a crucial component of U1 snRNP in mediating constitutive and alternative splicing events (de Francisco Amorim et al. 2018). LUC7 proteins are encoded by 3 genes in Arabidopsis (*AthLUC7A*, *AthLUC7B*, and *AthLUC7RL*). *luc7* triple mutants exhibit impaired development and hypersensitivity to ABA, cold, and salinity relative to the wild type. Both exon skipping and intron retention, particularly of terminal introns, are elevated in the *luc7* triple mutant. Strikingly, transcripts retaining LUC7-dependent introns in the *luc7* triple mutant escaped export from the nucleus, thereby avoiding NMD, which occurs in the cytoplasm. Interestingly, retention of the terminal intron is also enhanced in wild-type plants by cold and salinity suggesting an interplay between stress and LUC7 functionality. Finally, we note that a lncRNA has been shown to modulate alternative splicing regulators in plants (Bardou et al. 2014). The lncRNA, referred to as *AS COMPETITOR* lncRNA (or *ASCO*-lncRNA), interacts with a family of nuclear speckle RNA-binding proteins to control splicing patterns of several mRNA targets. The mechanism appears to involve competition between the *ASCO*-lncRNA and an alternative splicing mRNA target for binding these proteins and may be the tip of the iceberg for lncRNA modulation of alternative spicing (Kufel et al. 2022). Study of the plant spliceosome by single-molecule structurome methods (described later in this review) under unstressed and stressed conditions might give further insights into regulatory mechanisms.

### Scissors-like RNAs: ribozymes

Enzymes play exceptionally important roles in all forms of life. Enzymes have 4 key characteristics: (i) accelerating the rate of a chemical reaction; (ii) being highly specific for their substrates; (iii) undergoing multiple turnovers; and (iv) being unchanged in the reaction. The first enzyme discovered, in 1833, was diastase, which breaks down starch into maltose (Payen and Persoz 1883). For the next 150 yr, hundreds of enzymes were identified, and each was a protein. This paradigm was shifted in the early 1980s (Fig. 1) when Cech and Altman, working independently, demonstrated that RNA could be an enzyme. Cech dubbed RNAs that have characteristics of enzymes “ribozymes,” a portmanteau of “ribonucleic acid” and “enzyme.” The work by the Cech lab, conducted on Group I intron in the ciliated protozoan *Tetrahymena thermophila*, showed that splicing of the RNA intron could proceed without the spliceosome, or any proteins for that matter, under highly purified and reconstituted in vitro conditions (Kruger et al. 1982). This so-called self-splicing intron satisfied the first 2 characteristics of enzymes, those of rate acceleration, by ∼10^11^-fold over the uncatalyzed reaction, and of specificity, cleaving only a few phosphodiester bonds in a sea of millions of such bonds (Cech 1993). Although later work engineered a true enzyme version of this intron that carried out multiple turnover catalysis and was unchanged in the reaction in vitro (Zaug and Cech 1986), the naturally occurring Group I intron is not a true enzyme.

The work by Altman on RNase P, conducted in Gram-positive and -negative bacteria, showed that processing of the 5′-end of tRNA, described in the tRNA section above, could also proceed without protein, under highly purified in vitro conditions with elevated Mg^2+^ concentrations (Guerrier-Takada et al. 1983). Notably, RNase P naturally has all 4 characteristics of enzymes, including catalyzing multiple turnovers and being unchanged in the reaction. However, although Group I intron can operate without the assistance of proteins in vitro and in vivo (Zaug and Cech 1995), the RNA component of RNase P relies on the assistance of proteins in vivo, much like the ribosome and spliceosome as discussed above. Moreover, the number of proteins present in the RNase P complex increases with the complexity of the organism, from just 1 protein in bacteria to 10 proteins in humans (Walker and Engelke 2006). As one moves to plants, something altogether surprising happens: the RNA component is entirely lost and tRNA processing is carried out by 3 homologous protein enzymes dubbed “PRORP” for proteinaceous RNase P (Thomas et al. 2000; Gobert et al. 2010). Despite the prokaryotic origin of the chloroplast and mitochondria, both of these organelles utilize PRORP1, while PRORP2 and PRORP3 localize to the nucleus (Gobert et al. 2010). *prorp1* knockdown lines show defects in photosynthesis and drastic reductions in levels of mature plastid tRNA-Phe(GAA) and tRNA-Arg(ACG) (Zhou et al. 2015), while *prorp1* knockout lines are lethal (Gobert et al. 2010), as are *prop2 prop3* double mutants (Gutmann et al. 2012).

Group II introns comprise another large RNP complex with ribozyme characteristics. Group II introns, which are evolutionarily related to the spliceosomal RNAs in mechanism (see above), can operate as ribozymes without proteins in vitro, although they tend to associate with proteins in vivo (Lambowitz and Zimmerly 2011). Like Group I introns, Group II introns are not fully enzymatic in vivo, in that they do not catalyze multiple turnovers and are changed in the reaction. These ribozymes are characterized by 6 domains, DI–DVI, with DV having the active site and DVI containing the branchpoint A. Remarkably, Group II introns are mobile in that they can invade genomic DNA and insert themselves by a reverse splicing mechanism by way of an intron-encoded reverse transcriptase. This function helps explain why Group II introns are incapable of multiple turnovers (Lambowitz and Zimmerly 2011). Multiple crystal structures have been solved for self-splicing group I and II introns, and for RNA-based RNase P (Doherty and Doudna 2000; Mondragon 2013; Pyle 2016). These structures reveal an intricate network of secondary and tertiary interactions that help explain the catalytic mechanisms.

**Figure 7.**
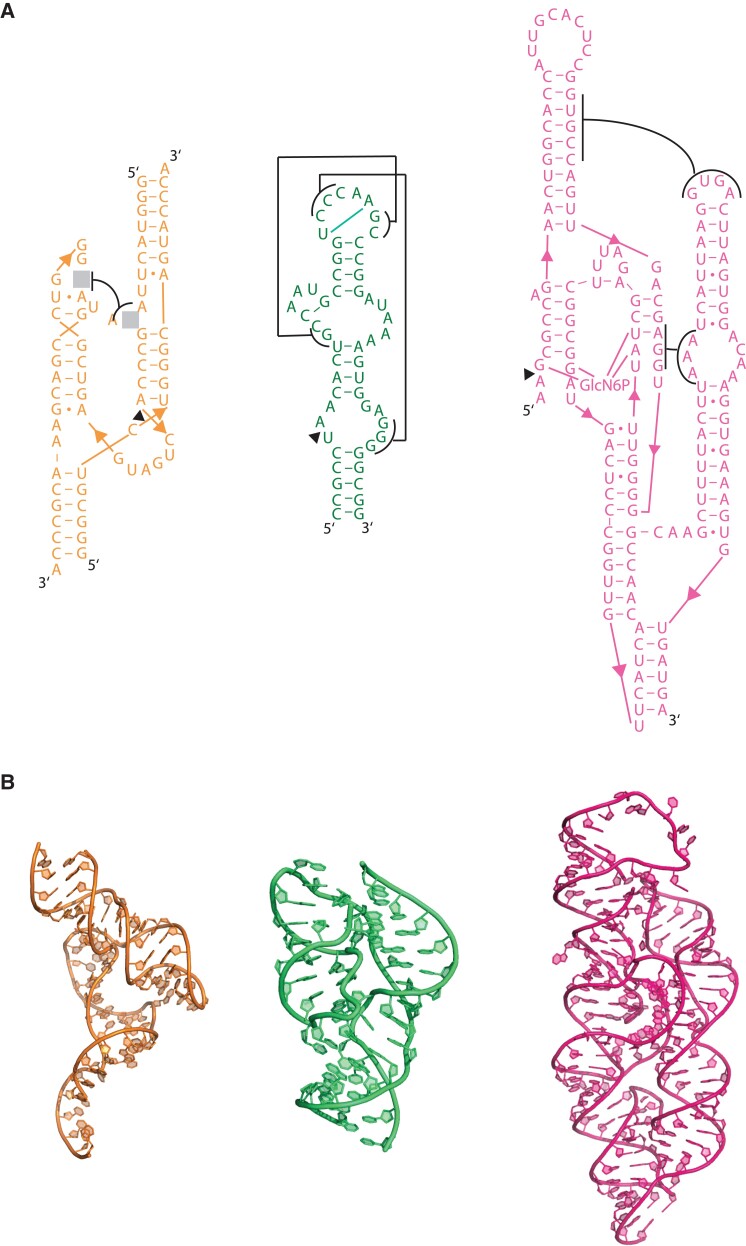
Ribozyme crystal structures. A) Secondary and B) tertiary structures for a hammerhead ribozyme that was laboratory evolved PDB ID: 5DI2 (Mir et al. 2015) (orange), twister ribozyme from rice PDB ID: 4OJI (Liu et al. 2014) (green), and *glmS* ribozyme from *Bacillus anthracis* PDB ID: 2NZ4 (Cochrane et al. 2007) (pink). These ribozymes are notable for their compact tertiary structures with a buried active site. The *glmS* ribozyme is also a GlcN6P-responsive riboswitch. The long-distance interactions depicted with black lines show double pseudoknots in twister and *glmS* ribozymes, and a single pseudoknot in the hammerhead ribozyme, which serve to compact and rigidify the structures. Adapted with permission from Seith et al. (2018), Fig. 2. Copyright 2018 American Chemical Society. Validation reports are available for these structures at the Protein Data Bank: https://www.rcsb.org/.

The above ribozyme examples are of relatively large RNAs, typically with lengths of 300 or more nucleotides. It is convenient, in fact, to separate ribozymes based on size. The smaller ribozymes, which typically range from ∼50 to 100 nts, encompass 10 subclasses: hammerhead, HDV, twister, glmS, twister sister, pistol, CPEB3, hairpin, pistol, and VS (Ferre-D’Amare and Scott 2010). Each subclass exhibits 1 or 2 pseudoknots. These pseudoknots compact the structure and create a relatively rigid active site; they also likely make the structure resistant to ribonucleases by virtue of their pseudoknot-containing folds (Akiyama et al. 2016). Representatives of 3 of the small ribozymes are provided in Fig. 7 and are notable for their compact tertiary structures with a buried active site. One aspect that separates the small and large ribozymes, besides their sizes, is their chemical mechanism. The large ribozymes leave 3′-OH and 5′-phosphate termini, whereas the small ribozymes leave 5′-OH and 2′,3′-cyclic phosphate termini. A main reason for this is that small ribozymes are not large enough to bind a cofactor to attack the phosphodiester bond and so instead use an internal 2′-OH. Using Shannon entropies, Szostak and co-workers elegantly showed that rate acceleration and complexity of mechanism are related to RNA length (Carothers et al. 2004), suggesting that the smaller ribozymes have simpler mechanisms, such as 1 instead of 2 chemical steps and no substrate binding instead of tRNA binding as in the case of RNase P.

Nowadays, once a new small ribozyme is discovered, its 3D structure and chemical mechanism are typically solved in less than a year. While much has been learned about the chemistry of ribozymes (Wilson and Lilley 2021), relatively little is known about their biology, with a few exceptions. For instance, the hammerhead ribozyme, as well as the HDV and hairpin ribozymes facilitate rolling circle replication of viroids, which make the most of their very compact genomes by housing their own cleavage agent (∼250–300 nt) (Flores et al. 2009). The ribozymes self-cleave to effectively cut the resulting concatemer product of rolling circle replication into unit-length monomers. Some of these ribozymes can undergo the reverse reaction and ligate, to convert the resulting monomers back into circles. Another instance where biology of a ribozyme is understood is in the glmS ribozyme (Fig. 7). This ribozyme is also a riboswitch (see below) and binds the sugar-phosphate cofactor glucosamine 6-phosphate (GlcN6P), which has diverse roles in phosphodiester bond cleavage (Bingaman et al. 2017). The consequence of cofactor-mediated ribozyme self-cleavage is mRNA degradation and less production of GlcN6P in a negative feedback loop.

With regard to the large ribozymes, originally identified from non-plant systems, self-splicing group I introns are narrowly distributed among plant lineages while Group II introns are more broadly distributed. Group I introns are found in several genes of green algae but in only a single chloroplastic gene of land plants (Kwon et al. 2020). Group I introns were reported in the *cox 1* (*cytochorome oxidase subunit 1*) mitochondrial gene of green algae and the nonvascular land plant, Marchantia (a liverwort), but were originally detected in only 1 flowering plant genus, Peperomia. With the rise of molecular phylogenetics, however, this sequence was found in 1998 to be present in *cox1* genes in 48 out of 341 angiosperms surveyed by Southern blot analysis (Cho et al. 1998), with still more instances identified in 2008 by sequence analysis (Sanchez-Puerta et al. 2008). The phylogenetically disjunct nature of the plant species that contain this intron suggests that this sequence was acquired numerous times by independent horizontal gene transfer events between plant species, seeded by a single original transfer from a fungus (Sanchez-Puerta et al. 2008).

Group II introns have been reported in plants. Plant mitochondria and chloroplasts harbor approximately 20 Group II introns each, although they have structural imperfections throughout DV and DVI, including loss of the bulged branchpoint A, that inhibit ribozyme activity (Barkan 2004; Lambowitz and Zimmerly 2011). The green alga *Euglena* has a remarkable ∼150 Group II-derived introns that lack various domains. *Trans*-acting RNAs and proteins, including the chloroplast factor MatK protein, likely assist splicing of this type of intron (Ems et al. 1995). It is remarkable that both RNase P and Group II ribozymes have either disappeared altogether or lost their catalytic activity in plants.

Of the 10 classes of small self-cleaving ribozymes, only 2—hammerhead and twister—have been reported in plants to date. Plant hammerhead ribozymes were first described in carnation (Daros and Flores 1995) and Arabidopsis (Przybilski et al. 2005). Bioinformatic searches based on combined sequence and structural patterns (Hammann and Westhof 2007; de la Pena and Garcia-Robles 2010) have since uncovered hammerhead ribozymes encoded in many plant genomes, from the green alga Chlamydomonas to vascular plants (de la Pena and Garcia-Robles 2010). Plant hammerhead ribozymes are often found within the paired long terminal repeats (LTRs) of retrotransposons. These retrotransposons are accordingly termed “retrozymes”; their ribozyme activity results in RNA fragments that can circularize, potentially providing a substrate for reverse transcription (RT) and reincorporation into the genome (Cervera et al. 2016). It has been hypothesized that viroids and satellite viruses that infect plants may in fact have arisen from these elements (de la Pena and Garcia-Robles 2010).

Biochemical study of plant hammerhead ribozymes has focused on the 2 hammerhead ribozymes encoded in the Arabidopsis genome: *Ara1* (At4g30860) and *Ara2* (At4g30870). *Ara1* expression was detected by RT-PCR in leaves, stems, and flowers, but not siliques, while *Ara2* expression was not detected. These ribozyme sequences were shown by in vitro assays to be catalytically active (Przybilski et al. 2005). However, as in most organisms, the biological function of ribozymes in plants remains unknown.

Twister ribozymes were first predicted bioinformatically by the Breaker research group and then experimentally verified as catalytically active (Roth et al. 2014). Among twister ribozymes with confirmed self-cleavage activity are 2 from rice, 1 of which has since had its structure solved by X-ray crystallography (Fig. 7) (Liu et al. 2014;Eiler et al. 2014). The report from Roth and colleagues predicted 6 more, to-date unverified, twister ribozyme candidates in rice. The variety of twister ribozymes in rice suggests potential biological importance. The functions of these small self-cleaving ribozymes remain unknown but twister regulation in vitro by pH, metabolites, and divalent cations (Roth et al. 2014; Messina and Bevilacqua 2018), and their presence in UTRs of rice genes annotated as disease-associated, may provide hints as to roles in cellular homeostasis or pathogen response.

## Paper-like RNAs I—conformational switching: RNA thermometers, riboswitches, and GQs

### RNA thermometers

Temperature is an important factor in physiology and development, especially in organisms (including plants) that do not thermoregulate (Mishra et al. 2018). RNA thermometers, commonly found in bacteria, are RNA structures present in 5′-UTRs or untranslated intergenic regions of operons that exhibit temperature-dependent alterations in conformation and thereby effect changes in gene expression. RNA thermometers typically consist of a hairpin that forms under cold conditions and melts under warm conditions. In the cold, the Shine–Dalgarno (SD) sequence, which is a bacterial and archaeal ribosome binding motif that base pairs with the anti-SD sequence in the 16S rRNA, is sequestered in the hairpin. Under warm conditions, hairpin unfolding renders the SD sequence available for ribosome binding, thus increasing translation. Melting of RNA thermometers present in the upstream UTRs of genes encoding protein chaperones increases their translation following heat shock. In pathogenic bacteria, e.g. *Listeria monocytogenes* and *Vibrio cholerae*, which are the causative agents of listeriosis and cholera, respectively, melting of RNA thermometers increases the translation of key transcriptional activators that control virulence factor expression, resulting in increased infectivity upon perception of mammalian host temperature (Johansson et al. 2002; Narberhaus 2010; Weber et al. 2014).

Two of the most common RNA thermometers are the 4U element and ROSE (repression of heat shock gene expression) thermometers. In the 4U element, the UUUU sequence base pairs with an AGGA SD sequence, with the 2 interior base pairs comprising G•U wobbles, which are thus particularly susceptible to melting. An alternative UCCU thermometer is present in the 5′-UTR of the cyanobacterial *Synechocyistis hsp17* gene, in which the G•U wobbles are replaced by canonical GC base pairs (Wagner et al. 2015). HSP17 chaperones photosynthetic proteins, thereby protecting photosynthetic function over a range of environmental temperatures (Wagner et al. 2015).

The ROSE element consists of 2–4 SLs (Righetti and Narberhaus 2014; Jolley et al. 2022). Originally, it was anticipated that the ROSE sequence UUGCU would interact with the AGGA in the SD sequence, creating a stem consisting of 3 canonical base pairs, a G•U wobble, and a single nucleotide G bulge. However, definitive NMR studies by Narberhaus and colleagues (Chowdhury et al. 2006) indicated that the G in this ROSE sequence actually forms a 2-hydrogen bond base pair with a G of the SD sequence, accompanied by a small internal loop. This report was important both as the first solution structure of an RNA thermometer and for the demonstration that temperature-induced melting required neither a protein cofactor nor a small molecule ligand, with the latter distinguishing RNA thermometers from riboswitches. Although we have touted the advantages of cryoEM, it should be noted that NMR, although limited to relatively short RNA sequences, also has advantages in structural investigations in that it avoids crystallization artefacts and can capture rapid changes in structure.

Intriguingly, although the classic RNA thermometers described above promote translation in response to temperature elevation, the first RNA thermometer identified, by Oppenheim and colleagues in 1989, regulates gene expression in the opposite fashion (Altuvia et al. 1989). This thermometer, present in the *c*III gene of the bacteriophage λ, exists in 2 conformations at equilibrium, like the bacterial RNA thermometers. However, based on in vitro structure probing with DMS (cf. Fig. 2B) and nucleases, heat in this case favors sequestration of the SD sequence and so opposes the formation of the translation initiation complex. The authors hypothesize that this occurs because the “open” conformation of the thermometer requires tertiary interactions that are destabilized by heat.

While RNA thermometers are widespread across bacteria, thermometers of this type appear to be scarce or absent in eukaryotes. Rather, studies in yeast and in plants (see the Structuromes section below) report whole-transcriptome restructuring during heat. In yeast, Chang and colleagues (Wan et al. 2012) extracted total RNA, refolded the RNA at 23 °C, and then subjected the samples to temperatures ranging from 30 °C to 75 °C. Samples were subsequently treated with RNase VI, which cleaves double-stranded RNA, followed by library preparation and sequencing of the retained single-stranded regions. Base pairs in “rock-like” RNAs (tRNA, rRNA, snoRNA, and snRNA) were observed to melt on average at higher temperatures than base pairs in mRNAs. Bacterial-like RNA thermometers were not described. Instead, within mRNAs, melting of motifs in 3′-UTRs was shown to increase access to the exosome, which degrades single-stranded RNAs from their 3′-end, resulting in reduced transcript abundance (Wan et al. 2012). This study, therefore, revealed temperature control of gene expression at the level of mRNA abundance, rather than at the level of translation as is the case for canonical bacterial thermometers.

In plants, the control of temperature-mediated morphological changes, so-called thermomorphogenesis, has been widely studied due to its impacts on plant growth and crop yield (Quint et al. 2016). The identification of a basic helix–loop–helix (bHLH) transcription factor *PHYTOCHROME INTERACTING FACTOR 4* (*PIF4*) was a milestone in understanding temperature signaling mechanisms in plants. PIF4 upregulates the expression of several key auxin biosynthesis genes at high temperatures, including *TRYPTOPHAN AMINOTRANSFERASE OF ARABIDOPSIS* (*TAA1*, also called *TRANSPORT INHIBITOR RESPONSE 2/TIR2*) and *SMALL AUXIN UP RNA* (*SAUR*) family genes that are known from T-DNA knockout studies to be required for plant development and heat-stimulated hypocotyl elongation (Yamada et al. 2009; Franklin et al. 2011).

**Figure 8.**
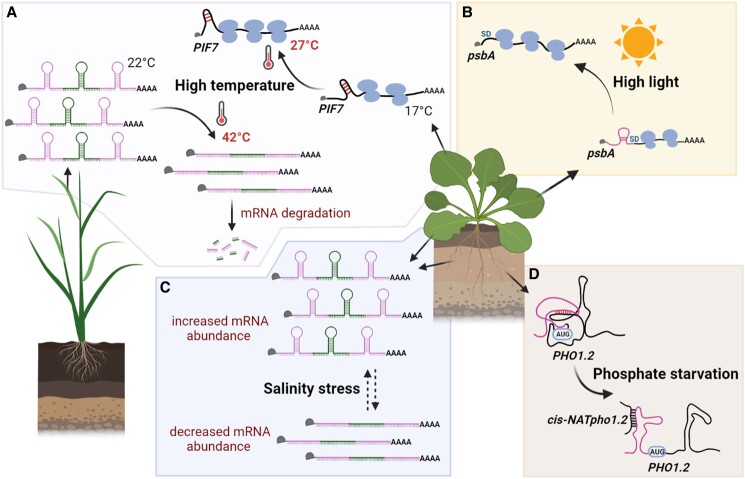
Schematic of RNA structure–function relationships in abiotic stress response. A) Upper, right portion: under warmer daytime temperatures, the RNA hairpin within the 5′ UTR of *PIF7* attains a more-relaxed hairpin conformation (weaker RNA base pairing) than at lower nighttime temperatures, resulting in increased *PIF7* translation (Chung et al. 2020). Lower, left portion: in rice seedlings, acute heat shock leads to genome-wide mRNA structural melting and consequently mRNA degradation (Su et al. 2018). B) High light triggers unfolding of an SD-sequestering hairpin in the 5′ UTR of the *psbA* mRNA, which consequently increases *psbA* translation (Gawronski et al. 2021). C) Salt, likely acting indirectly (dashed lines), appears to unfold some transcripts (higher reactivity to the structure probing chemical DMS [cf. Figure 1B]) with correlated decreases in their abundance, and fold other transcripts (lower reactivity to DMS) with correlated increases in their abundance (Tack et al. 2020). D) In response to phosphate starvation, an intermolecular sense to antisense interaction between *PHO1.2* and *cis-NAT PHO1.2* rearranges the RNA conformation, leading to the enhancement of *PHO1.2* translation (Reis et al. 2021).

*PHYTOCHROME INTERACTING FACTOR 7* (*PIF7*) also mediates thermomorphogenesis, as knockout of either *PIF4* or *PIF7* impairs heat-stimulated hypocotyl and petiole elongation. Interestingly, a recent study revealed that the enhanced translation of *PIF7*under warm temperatures is mediated by temperature-responsive structural changes in the 5′-UTR of the *PIF7* mRNA (Chung et al. 2020). In vitro translation of a luciferase reporter gene fused to the *PIF7* 5′-UTR was reduced when a hairpin structure within the UTR was eliminated. Additionally, heat-enhancement of translation was also disrupted upon stabilization of the hairpin by substituting in stronger C-G base pairs. These results suggested that temperature-dependent conformational flexibility of the hairpin, which was confirmed in circular dichroism studies, is required for enhanced *PIF7* translation at elevated temperatures (Fig. 8A). In the same study, temperature-dependent translational enhancement by partial melting of a structured hairpin was also implicated for some other key regulators in thermomorphogenesis, such as in mRNAs of the *WRKY22* transcription factor and *HEAT SHOCK FACTOR A2* (*HSFA2*), suggesting a conserved temperature-responsive translational regulatory machinery at the RNA structural level that allows plants to acclimate to temperature changes (Chung et al. 2020). However, as described in a later section, our analysis (Su et al. 2018) of the rice transcriptome following heat shock did not find evidence for microbial-type RNA thermometers, with numerous mRNAs showing restructuring in response to heat rather than simple 5′-UTR melting.

### Riboswitches

Allostery, in which interaction with a cofactor or other molecule regulates enzyme activity, has been a familiar concept in protein biochemistry since the 1960s (Liu and Nussinov 2016). Demonstration that an analogous mechanism is executed by RNA structures named “riboswitches,” in which RNA–ligand interactions control gene expression at the level of transcription or translation, came several decades later. Pioneering work from Grundy and Henkin (Grundy and Henkin 1993) described the T-box riboswitches, which preferentially base pair with uncharged tRNAs, thereby inducing expression of genes involved in tRNA charging (i.e. aminoacylation) of the cognate amino acid, as well as synthesis of the amino acid. Charged tRNAs fail to activate the riboswitch due to steric clash with the bound amino acid. This is a particularly unique example of positive regulation by a riboswitch; as described below many riboswitches function as negative feedback regulators of ligand synthesis. Many of the riboswitches we describe below also exhibit novel modes of molecular recognition of their small molecule ligands, as opposed to the well-known paradigm of nucleic acid base pairing.

The discovery of riboswitches was presaged by research on transcription termination in bacteria, particularly in the *trp* (tryptophan) operon. Research from the Yanofsky lab on a series of deletion mutants of *trp* demonstrated that a structure in the 5′-leader sequence which they dubbed the terminator, consisting of a very stable G-C-rich hairpin followed by a series of Us that make unstable base pairs with templating dAs (Stroynowski and Yanofsky 1982), causes stalling of RNA polymerase that ultimately results in transcript termination. In deletion mutants wherein an alternative hairpin structure, the antiterminator, is favored to form upstream, the terminator cannot fold and transcription proceeds (Stroynowski and Yanofsky 1982; Stroynowski et al. 1983). This research introduced the idea of RNA conformational switching as a regulator of gene expression. What would cause switching in vivo was not known initially, but subsequent research demonstrated that, in the case of the *trp* operon, regulation is mediated by the RNA-binding protein, TRAP (Babitzke and Yanofsky 1993). In parallel with this research were studies from the Gold, Szostak, and Joyce groups in which particular RNA sequences were engineered or artificially evolved to bind small molecule ligands (Ellington and Szostak 1990; Tuerk and Gold 1990) and in some cases also self-cleave (Robertson and Joyce 1990), i.e. show allosteric regulation of activity (Tang and Breaker 1997). These 2 research strands were woven together in 2002 (Fig. 1), with the first discoveries by the Breaker and Nudler laboratories that direct transcript-binding of small molecules can induce RNA structural changes that regulate gene expression without the aid of protein or nucleic acid partners (Mironov et al. 2002; Nahvi et al. 2002; Winkler et al. 2002).

Nudler and colleagues used an in vitro transcription assay to demonstrate that the thiamine (vitamin B1) gene of *Bacillus subtilis* is subject to thiamine pyrophosphate (TPP)-induced transcription termination (Mironov et al. 2002). Breaker and colleagues (Nahvi et al. 2002; Winkler et al. 2002) demonstrated that the 5′-UTR of the thiamine gene of *E. coli* undergoes structural rearrangement in vitro in the presence of thiamine derivatives, particularly TPP. Importantly, these effects were observed in the absence of any proteins and thus rigorously attributable to metabolite binding. Predicted RNA structures were consistent with TPP-induced sequestration into a hairpin of the SD sequence, which is required to be unpaired for ribosome binding in prokaryotes. In vivo reporter gene assays using translational fusion constructs with the beta-galactosidase gene showed that mutation of the ligand-binding structure led to loss of suppression of beta-galactosidase activity by thiamine derivates. These results indicated a negative feedback loop wherein high levels of a metabolite arising from the activity of the encoded enzyme suppress the expression of that enzyme.

**Figure 9.**
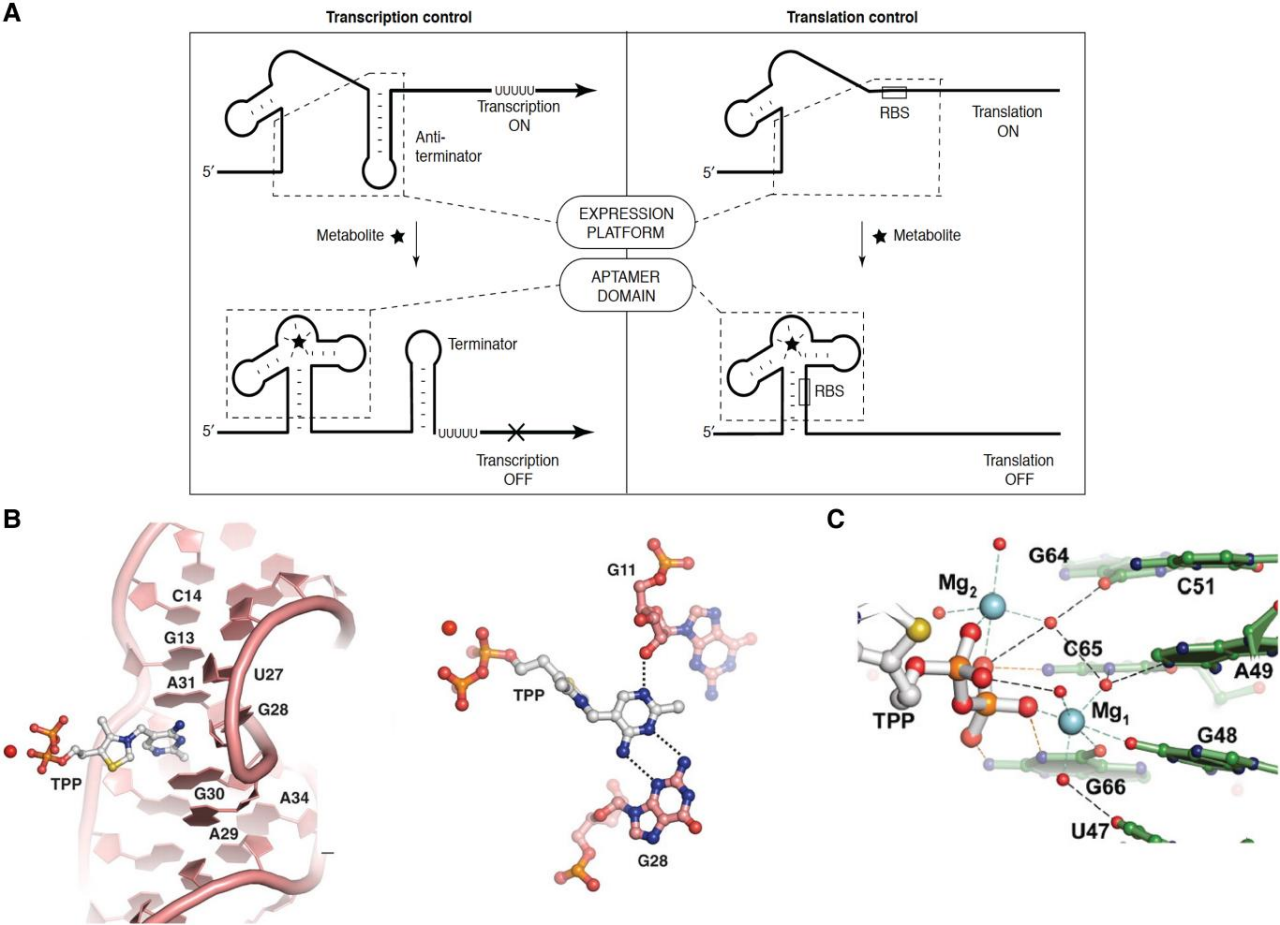
Mechanisms of riboswitch-mediated gene regulation. A) Riboswitches can operate under transcription control (left) or translation control (right) and can be divided into an Aptamer Domain and an Expression Platform. This scheme is for the metabolite turning OFF transcription or translation, although ON riboswitches are also known. In transcription control, an antiterminator forms that is mutually exclusive with the terminator owing to sharing nucleotides. Upon binding of the metabolite, depicted generically as a star, to the Aptamer Domain, nucleotides in the antiterminator are sequestered into the folded aptamer and the terminator forms, which is stable and has a series of Us that make unstable base pairs with templating dAs and terminates transcription. In translation control, the ribosome binding site (RBS), or SD site in the case of bacteria, is exposed and translation is ON. Upon binding of the metabolite to the aptamer domain, the RBS is sequestered into the folded aptamer and translation is turned OFF. Reprinted with permission from Tucker and Breaker (2005). Copyright 2005 Elsevier. B, C) Molecular recognition of TPP by the riboswitch from Arabidopsis (Thore et al. 2006; Thore et al. 2008). The TPP metabolite is bound in a modular fashion, with nitrogen atoms in blue, oxygen atoms in red, and carbon atoms in a specific color. B) Recognition of the thiamine (white) portion of TPP. There is extensive molecular recognition of the metabolite provided by diverse faces of riboswitch nucleobases (salmon) PDB ID: 2CKY (Thore et al. 2006). Copyright 2006 AAAS. C) Recognition of the pyrophosphate portion of TPP. There is extensive recognition by 2 Mg^2+^ ions. Note that panel C uses the refined crystal structure that led to a new conformation of the pyrophosphate moiety PDB ID: 3D2G (Thore et al. 2008). Copyright 2018 American Chemical Society. Validation reports are available for these structures at the Protein Data Bank: https://www.rcsb.org/.

The above work was just the beginning, as numerous classes of both transcription terminating and translational attenuating riboswitches have since been documented (Breaker 2022). At their core, riboswitches consist of 2 domains arising from separate or overlapping sequences: the aptamer domain, where metabolite binding occurs, and the expression platform, which controls gene expression (Fig. 9A). The aptamer domain is known for its compact tertiary structure, driven by pseudoknot formation as in ribozymes, and its exquisitely specific molecular recognition. Crystal structures have been solved for almost all known riboswitch aptamer domains, with the TPP riboswitch from Arabidopsis shown in Fig. 9, B and C as a representative one. This structure is notable for the modular recognition of the ligand, with 1 set of nucleobases interacting with the thiamine portion of TPP (Fig. 9B) and another set of nucleobases along with 2 Mg^2+^ ions interacting with the pyrophosphate moiety of TPP (Fig. 9C). Nearly every functional group of TPP that can hydrogen bond does. This makes one appreciate the potential for other plant metabolites rich in hydrogen bond donors and acceptors to be specifically bound by RNA. Because each aptameric domain has a unique structure that enables specific molecular recognition of its cognate metabolite, it has been possible to use sequence and structure descriptors of the aptamer domain to bioinformatically search for new instances of known riboswitches (Barrick et al. 2004; McCown et al. 2017). Although the ligands for some bioinformatically predicted riboswitches remain unknown (Sherlock and Breaker 2020), as of 2022 over 50 classes of validated riboswitches have been described in prokaryotes. Riboswitch-regulated genes function in many fundamental biochemical pathways related to C, N, and S metabolism (Breaker 2022). Moreover, riboswitches that sense ions (Mg^2+^, Mn^2+^, F^−^, Ni-Co, Na^+^) have also been documented. In addition, as noted by Breaker and colleagues (McCown et al. 2017), many riboswitch ligands are RNA-derived compounds, e.g. TPP, flavin mononucleotide (FMN), and cyclic-diGMP. This phenomenon may reflect the intrinsic ability of RNA to perform intermolecular base pairing and is perhaps indicative of the importance of RNA-based compounds in the RNA World. Other ligands include cofactors, vitamins, and amino acids, and encompass functional groups that hydrogen bond well with RNA such as amines, carbonyls, and hydroxyls. This suggests that RNA is an adept small molecular binder and that many riboswitches may remain undiscovered, as suggested recently by Breaker (McCown et al. 2017; Kavita and Breaker 2022).

While, as described above, riboswitches are common in bacteria, the same does not appear to be true in the other 2 domains of life, perhaps because the path of evolution selected for proteins as the dominant allosteric regulators in those organisms, as supported by proteinaceous PRORP RNase P and non-catalytic group II introns in plants. There are only a few riboswitch classes known so far in archaea, and just 1 eukaryotic riboswitch class, TPP, which has been detected in some fungi, plants, protists, and a single metazoan (McCown et al. 2017).

As early as 2003 (Sudarsan et al. 2003), the Breaker team reported that predicted transcriptomes of several fungal and plant species, including Arabidopsis, contained motifs that matched the consensus sequence and structure for the TPP aptamer domain. TPP-induced conformational switching of the Arabidopsis sequence was confirmed in vitro by in-line probing methods, in which spontaneous cleavage of the backbone of the RNA, which is influenced by structure, is read out in the size-dependent migration of ^32^P-labelled cleavage products in gel electrophoresis. The fungal TPP riboswitch is located in an intron within the 5′-UTR of the *thiA* gene, which encodes an enzyme in the pathway of thiamine synthesis. In the fungus *Aspergillus oryzae*, 5′-UTR splicing required for gene expression was shown to be inhibited by thiamine (Kubodera et al. 2003), constituting a third mechanism of negative feedback gene regulation by riboswitches. In the fungus *Neurospora crassa*, the TPP riboswitch is present in 5′-UTR introns of 3 genes, where it regulates alternative splicing, with TPP repressing translation of 2 of these by promoting the formation of a splice variant that retains 1 or 2 upstream ORFs that then compete with the main ORF for translation, illustrating a fourth riboswitch regulatory mechanism.

The TPP riboswitch of plants illustrates yet a fifth mechanism of riboswitch regulation (Figs. 3B, 9, B and C). The plant TPP riboswitch is located in the 3′-UTR of the thiamine biosynthesis gene, *THIAMINE C SYNTHASE* (*THIC*). As elegantly revealed by PCR-based and Northern blot analyses of *THIC* transcript composition and length (Bocobza et al. 2007; Wachter et al. 2007), TPP binding results in changes to the length of the *THIC* transcript via inducing both alternative splicing and end processing events. When TPP concentrations are low, the interaction of the aptamer domain with an upstream 5′-splice site within the 3′-UTR prevents splicing. Within the consequently retained intron is a cleavage site that results in a short transcript that is then polyadenylated and highly expressed. Conversely, when TPP concentrations are high, the aptamer sequence no longer base pairs with the 5′-splice site, which then becomes available for intron excision, resulting in loss of the processing site. Transcription then extends up to ∼1 kb until cryptic 3′-end processing sites are encountered; these longer transcripts are more susceptible to degradation, as confirmed by assays using transcriptional inhibitors (Bocobza et al. 2007), resulting in decreased transcript abundance.

This TPP aptamer sequence is highly conserved across diverse plant taxa, including moss, lycophytes, gymnosperms, and both monocot and dicot species of angiosperms. However, angiosperms and some gymnosperms have lost a second TPP riboswitch, present in the *THI1* gene of the other plant taxa (Bocobza et al. 2007). Interestingly, in the green algae Chlamydomonas and Volvox, a TPP riboswitch that regulates splicing is found in an intron within the coding region, rather than within either UTR (Croft et al. 2007).

Thiamine (vitamin B1) is a precursor for the formation of TPP, an important cofactor for enzymes in the TCA cycle and pentose phosphate pathway. Thiamine levels rise under abiotic stresses associated with ROS production (supraoptimal light, cold, salinity, paraquat application), and exogenous thiamine application reduces H_2_O_2_ levels induced by paraquat, possibly by increasing NADPH production from the pentose phosphate pathway (Tunc-Ozdemir et al. 2009). Thiamine, via unknown mechanisms, is also implicated in the induction of pathogenesis-related (PR) genes during pathogen defense (Ahn et al. 2005), and in appropriate responses to changes in photoperiod (Rosado-Souza et al. 2019). However, the direct role of the TPP riboswitch in these phenomena remains to be studied.

### G-quadruplexes

**Figure 10.**
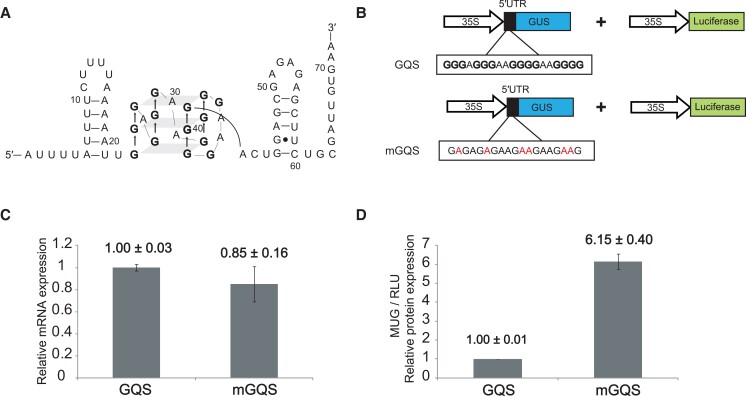
Inhibition of translation by the formation of a 5′-UTR GQ structure. A) The GQ-forming sequence of G_3_AG_3_AAG_4_AAG_4_ found in the 5′ UTR of the *ATR* gene in Arabidopsis. B) Schematic of constructs used in this transient expression reporter assay to test the effect of the GQ on transcription and translation. The GQS construct contains the complete native *ATR* 5′ UTR, while in the mGQS construct select Gs are mutated to As (red), which prevents GQ formation. Shown are normalized C) GUS/luciferase mRNA abundance as assessed by qRT-PCR, and D) protein expression as assessed by enzyme activity. Based on the comparison of results from GQS and mGQS constructs, translation, but not transcription, is repressed by the GQ structure. Reproduced from Kwok et al. (2015).

Tandem repeats of Gs can lead to a structure known as a G-quadruplex (GQ), which involves pairing of 4 different Gs in a planar array called a “G-quartet” (Fig. 10A). These structures form in ssRNA and ssDNA, which is present at the end of chromosomes as telomeres. All known GQs have at least 2 tiers of quartets and often conform to the motif (*G_x_L_y_*)_4_, where *L* is a loop nucleotide and *x* ≥ 2 and *y* is typically ≤7. For instance, the sequence 5′-GGAGGAGGAGG can fold into a 2-tier parallel-stranded quadruplex, in which G1, G4, G7, and G10 form 1 quartet, and G2, G5, G8, and G11 form the second quartet, which stacks on the first one. Biological sequences can also possess more complex but still contiguous motifs containing irregular repeats of Gs and Ls. Additionally, GQs can form from noncontiguous stretches of Gs, especially as elucidated in structures of dye-binding aptamers (Banco and Ferre-D’Amare 2021), and provide exciting extensions of GQs, although at present they are difficult to predict. The GQ is stabilized by K^+^ ions; although Na^+^ ions can also provide some stability they will be of low concentration in non-halophyte plants. The K^+^ ions reside in the center of the quartet and interact with the quartet above and below. Notably, the quadruplex structure can come from 1 sequence folding back on itself, as in (G_2_A)_4_, or by the association of up to 4 different strands in which case (*G_x_L_y_*)*_n_* can form with *n* < 4, making this interaction plausible in the concentrated environment of a phase-separated liquid droplet (Bevilacqua et al. 2022).

In RNA, the GQ is often found in the 5′-UTR (Fig. 10A) where it can inhibit gene expression by blocking transcription or translational apparatuses or by sequestering essential regions of the transcript. The extent to which GQ form in vivo has been controversial. While there is compelling evidence for GQ formation in bacteria, it has been suggested that in eukaryotes, helicases maintain GQ as unwound sequences (Guo and Bartel 2016). However, recent studies in plants (see below) suggest otherwise.

Perhaps the first study that suggested GQ formation in plants was a bioinformatic analysis from our labs (S.M.A. and P.C.B.; Mullen et al. 2010). We searched for tandem stretches of G residues and found ∼1,200 possible GQs with a G3 repeat and over 40,000 with a G2 repeat. The former class is enriched in intergenic regions while the latter is found in genic regions. GQ sequences are over-represented in transcripts encoding enzymes but underrepresented in structural RNAs such as tRNAs and rRNAs. Interestingly, genes responsive to drought stress, which alters K^+^ levels, are enriched in GQ sequences. This seminal bioinformatic study suggested that GQ sequences could regulate the expression of key proteins during stress response. In the last decade, additional studies have provided further support for GQ formation in plants.

A functional role of GQ in translational regulation in vivo was implicated in our early study (Kwok et al. 2015) on an Arabidopsis mRNA encoding the protein kinase, ATAXIA TELANGIECTASIA-MUTATED AND RAD3-RELATED (ATR), which is required for cell-cycle regulation and telomere maintenance in DNA damage and repair processes (Amiard et al. 2011). We first observed that a GQ in the 5′-UTR of *ATR* forms in vitro with high thermostability. This GQ appears to repress translation in living plant cells based on transient reporter gene assays (Fig. 10, B–D). The GUS reporter gene was fused to either the GQ or a GQ mutant (6 Gs in the GQ were mutated to As, resulting in the disruption of GQ formation), and relative RNA and protein expression were determined by comparing the normalized GUS/luciferase mRNA abundance or GUS/luciferase activity between the 2 constructs. While transcript abundance was similar for GQ vs. GQmut, GUS enzymatic activity was increased in the GQmut, suggesting that this GQ participates in translational repression (Kwok et al. 2015).

More recently, in vivo SHALiPE-seq (Selective 2′-hydroxyl acylation with lithium ion-based primer extension sequencing; Table 1) (Yang et al. 2020b) has been applied to Arabidopsis to query the presence of GQs in vivo and transcriptome-wide. Given the chemical property that the last G residues of G-tracts in folded GQ are preferentially modified by 2-methylnicotinic acid imidazolide (NAI), the SHALiPE-seq method can identify structured GQ positions. Several hundred GQs were found by this method, suggesting that, at least in Arabidopsis, GQs do form in vivo, albeit at a much lower frequency than predicted from computational analysis, at least as detected by this method.

In addition, several studies have functionally implicated GQs in root development and abiotic stress response. Roots serve a variety of functions, including water and nutrient uptake, nutrient storage, and plant anchorage. Several recent studies have implicated GQs in the regulation of genes involved in root development. For example, *HIRD11* mRNA (At1G54410) encodes a KS-type dehydrin and contains a G2 (with 2 G-quartets) GQ in its 3′-UTR, as recently identified from an in vivo SHALiPE-Seq structure profiling study (Yang et al. 2020b). *hird11* GQ mutants exhibit shorter roots relative to wild type (Fig. 3C). The GQ structure was shown to regulate *HIRD11* transcript abundance and root morphology through a mutagenesis study of GQ (mutGQ), in which the 4 G residues were substituted by A-residues, which exclude the possibility of intramolecular GQ folding. Higher *HIRD11* transcript abundance and longer roots were observed in the mutGQ *hird11* transgenic plants than in wtGQ *hird11* plants, suggesting an important role of GQ in *HIRD11* posttranscriptional regulation and root development.

GQ-mediated translational regulation in phloem development in the root has been revealed by the identification of a RanBP2-type zinc-finger protein JULGI (JUL1) and its RNA targets, the 5′-UTRs of the *SUPPRESSOR OF MAX2 1-LIKE4/5* (*SMXL4/5*) mRNAs (Fig. 3D) (Cho et al. 2018). JUL1 protein induces GQ formation upon binding, which suppresses *SMXL4/5* translation and hence restricts phloem differentiation—SMXL4/5 are pivotal for phloem development based on this study as well as an earlier T-DNA knockout mutant study, in which the root apical meristem size of 10-d-old *smxl4/smxl5* seedlings was significantly decreased relative to wild type (Wallner et al. 2017). Notably, from a 5,6-carboxyfluorescein diacetate (CFDA) fluorescence tracking assay of phloem transport, *JUL1* RNAi transgenic plants exhibit enhanced phloem transport capacity over wild-type, leading to higher sink strength, bigger seed size and heavier grain weight. The transcript abundance of *SMXL4/5* is upregulated in the *JUL1* RNAi mutants as well (Cho et al. 2018).

The root endodermis is a barrier that separates the inner vascular tissue of the phloem and xylem from the outer cortex, and is structurally crucial for selective nutrient uptake, as well as avoiding backflow of water from the vascular system (Tajima 2021). *SHORT ROOT* (*SHR*) is a GRAS-type (***G****IBBERELLIC ACID INSENSITIVE, **R**EPRESSOR OF G**A**1, and **S**CARECROW*) transcription factor that is a pivotal player in root development. Overexpression of *SHR* with a 35S constitutive promoter results in extra cell layers in the root cortex, which show evidence of mis-specification as vascular tissue (Helariutta et al. 2000). Notably, single-molecule RNA FISH (smFISH) localization revealed that *SHR* RNA accumulates in cytosolic structures reminiscent of phase-separated compartments. *SHR* contains a GQ that can promote RNA-driven liquid–liquid phase separation (LLPS) in vitro (Zhang et al. 2019) (Fig. 3E). Given the importance of LLPS for cellular functions in modulating biochemical complexes and biological reactions temporally and spatially (Hyman et al. 2014), *SHR-GQ* structure change could be a mechanism that could tune the translation efficiency of *SHR* mRNA under stress and thus modulate root development. For example, cytosolic K^+^ concentrations in plants are known to differ between different developmental stages and stress conditions (e.g. salinity, osmotic stress, and drought) (Levi et al. 2011; Ragel et al. 2019); stress-induced higher K^+^ concentrations could promote GQ-triggered RNA-driven phase separation in cells, which might protect or store *SHR* mRNA that is required for root development.

Stress granules and P-bodies exemplify LLPS, wherein specific RNA-binding proteins could direct their RNA partners to the condensate, or vice versa. Sequestration in stress granules can render RNA translationally inactive while protecting it from degradation (Chantarachot and Bailey-Serres 2018). LLPS (Williams et al. 2022) and aggregation (Williams et al. 2021) can be mediated by GQs, perhaps because they can drive multimeric strand formation as mentioned above (Bevilacqua et al. 2022). Recent studies also indicate that flanking sequences can disrupt GQ formation, as can CTP (Williams et al. 2022). Recently, a mammalian stress granule marker protein, Ras GTPase-activating protein-binding protein 1 (G3BP1), was found as a direct interacting partner with a GQ from a fluorescence anisotropy assay, with the observed structure-selective binding affinity suggesting GQ's function in mediating RNA stability in stress granules (He et al. 2021). In fact, a novel function of plant GQ in mitigating mRNA decay during cold adaptation has also been proposed (Yang et al. 2022b). In monitoring the decay rate of mRNAs post-cold exposure, the cold-associated formation of GQs in 3′-UTRs (but not in 5′-UTRs or CDSs) was correlated with higher mRNA stability, while the mRNA decay rate was greater among GQ-lacking transcripts after low-temperature treatment.

Regarding the versatile functions of GQs in RNA stability and protein translation, and the possibility of their regulation by helicases, it is worth mentioning that the first plant-type GQ helicases have been identified recently by a GQ-binding protein pull-down mass spectrometric study (Wang et al. 2022). The structural unwinding capacity among these GQ helicases was validated by a microscale thermophoresis assay. In addition to the newly identified plant-type GQ helicases, many GQ interacting proteins were identified in this study, with biological functions in pre-mRNA splicing and translation regulation. Given the structural dynamics of GQs following abiotic stress stimuli, these findings provide new leads for understanding GQs’ functions in plants and other organisms.

## Paper-like RNAs II: many conformations

### History and methods of in vivo structure probing

RNA structure in the living cell is influenced by myriad factors: the distinct environments of the nucleus, cytosol, and RNA-containing organelles; a panoply of RNA-binding proteins that can directly (e.g. RNA helicases) or indirectly (e.g. by binding and sequestering transcript regions) alter structure; and the possibility of co-transcriptional folding. This complexity dictates that test tube simulations of in vivo conditions will fall short of capturing the true in vivo structures of all but the most rock-like of RNAs. While in silico modeling, particularly of the most thermodynamically stable structure of an RNA sequence, is quite advanced, RNAs in vivo can become trapped in alternative structures, never reaching the thermodynamic minimum (Zemora and Waldsich 2010). This is especially true for folding during transcription (Bushhouse et al. 2022). Accordingly, elucidation of RNA structure in vivo remains one of the great challenges of the field. It is exciting that major methodological advances toward solving this problem have been made over the last decade, some of which are summarized below, with a focus on those that have been applied to plant systems. In particular, the first transcriptome-wide investigations of RNA structure—the so-called RNA structurome—were published in 2014 (Fig. 1) (Ding et al. 2014; Rouskin et al. 2014). These accomplishments were built on several decades of methods development for probing the structures of individual RNA species, as described below and noted in Fig. 1.

In vitro methods of probing the structures of individual RNA species include NMR, crystallography, and cryo-EM. These low throughput methods are complemented by methods in which the structure of an RNA species is read out on gels or via capillary electrophoresis, using either end-labeling of the RNA or RT. These approaches are based on structurally mediated sensitivity to (i) spontaneous degradation (in-line probing); (ii) nucleases that specifically target single-stranded or double-stranded regions; or (iii) chemical probes of RNA structure. With the exception of hydroxyl radical footprinting of cell cultures, in which accessible regions of the RNA are oxidized in vivo and the resultant cleavage patterns then read out (Chea and Jones 2018), only the chemical probing strategy is amenable to in vivo application.

Chemicals that covalently modify RNA were described as early as the 1960s (reviewed in [Ehresmann et al. 1987]). The first report on the use of such chemicals in vitro to query RNA structure was in 1980 (Fig. 1), from Peattie and Gilbert (1980). In that study, DMS, which covalently modifies the N1 of A and the N3 of C when these residues are not engaged in base pairing (Fig. 2B), as well as the N7 of guanosines, was applied, as was diethyl pyrocarbonate, which detects stacking of As, along with gel-readout to query the secondary and tertiary structure of end-labeled yeast tRNA^Phe^ upon progressive denaturation.

DMS readily permeates cell membranes, and its first in vivo use for RNA was to probe the structure of the 5′-leader sequence of the *rplJ* operon (Climie and Friesen 1988). Another early study that employed DMS-based in vivo chemical probing of an mRNA was performed in a plant system, wherein the structures of the 18S rRNA and the highly abundant Rubisco SSU (encoded in the nucleus) mRNA of soybean were probed (Senecoff and Meagher 1992). The 18S rRNA structure was shown to agree with the phylogenetic structure, while the SSU mRNA was shown not to adopt the lowest free energy state, as we now know is true for many transcripts in vivo. Given that portions of prokaryotic transcripts are often highly structured (Ritchey et al. 2020), the structures of other mRNAs encoded by the chloroplast genome were also of interest. In 1999, DMS probing in the green alga, Chlamydomonas, was employed by Stern and colleagues (Higgs et al. 1999) to identify structural elements in the 5′-UTR of the *petD* gene, which encodes subunit IV of the cytochrome b(6)f complex. These DMS results, combined with in vitro structure probing with nucleases, suggested the presence of 2 hairpins that promoted translation, the upstream of which also was required for RNA stability. The 5′-UTR of *rbcL*, which encodes the LSU of Rubisco, similarly has a 5′-upstream hairpin, identified by DMS probing and structure prediction (Suay et al. 2005). Although this hairpin was also found to be essential for RNA stability, as read out by Northern blot analysis of transcription of a fused GUS reporter gene, comparable hairpins of different sequences failed to stabilize the transcript, leading the authors to posit that in this instance the hairpin is a target for an RNA-binding protein that protects against transcript degradation.

Since those early days of in vivo structure probing, 2 major changes have been implemented: the toolkit for structure probing, both in vitro and in vivo, has been developed and expanded (Bevilacqua and Assmann 2018), and probing has been expanded to the entire transcriptome (Table 1). Recently, glyoxal, which targets the Watson–Crick face of Gs that populate their anionic state, and the 1-ethyl-3-(3-dimethylaminoproply) carbodiimide (EDC), which targets the Watson–Crick face of accessible Us and Gs, have shown promise as in vivo probes of RNA structure (Mitchell et al. 2018; Mitchell et al. 2019; Wang et al. 2019a). This is an important advance because it enables probing of the Watson–Crick faces of all 4 bases in vivo for the first time (Fig. 2B). SHAPEreagents have been developed for in vitro and in vivo use, and these can also modify all 4 nucleotides, in this case by acylating the 2′-hydroxyl (2′-OH) of the sugar-phosphate backbone in “flexible” RNA regions (Fig. 2B). In particular, 2-methylnicotinic acid imidazolide (NAI) and 2-methyl-3-furoic acid imidazolide are SHAPE reagents with improved membrane penetrability (Spitale et al. 2013).

**Figure 11.**
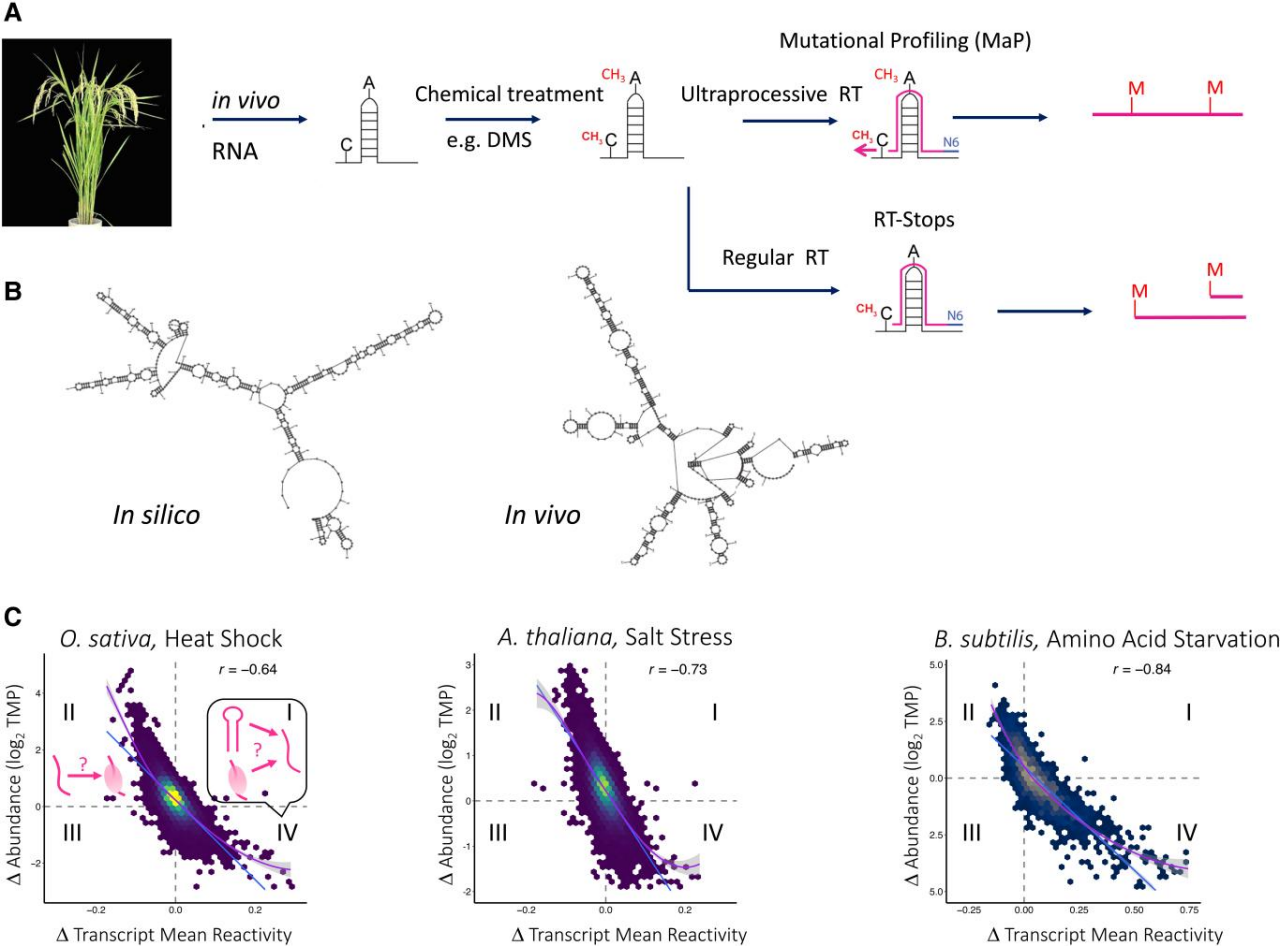
Structurome approaches and observations for RNA folding transcriptome-wide. A) Approach for mutational profiling (MaP) vs. RT stops. Treatment with DMS results in methylation of exposed A and C residues, which are detected as mutations in a single read in MaP (M; top) or as a series of truncated transcripts in RT stops (M; bottom). B) Large differences in predicted and in vivo determined structures for *RARE-COLD-INDUCIBLE 2A* (*RCI2A*; At3g05880) (Ding et al. 2014). C) Inverse relationship of change in chemical probe reactivity and change in transcript abundance for 3 different organisms under 3 different stresses: *O. sativia* under heat shock (Su et al. 2018), *A. thaliana* under salt stress (Tack et al. 2020), and *B. subtilis* under amino acid starvation (Ritchey et al. 2020).

Another major challenge has been to move from in vivo studies of single RNA species to obtaining structural data on the entirety of the transcriptome of a living system, i.e. the in vivo “structurome.” This challenge was solved in 2014 (Fig. 1), when in vivo genome-wide RNA structure probing approaches were first developed using DMS by 2 independent research teams (Ding et al. 2014; Rouskin et al. 2014). The Structure-seq method (Fig. 11A) was developed by our laboratories. Structure-seq uses a random hexamer primer for RNA RT, and the derived cDNAs are then subjected to adapter ligation and PCR amplification. DMS-modified positions in RNA block RT and thus generate prematurely terminated cDNAs. The resultant cDNA library is examined by Illumina sequencing followed by DMS reactivity calculation and RNA structure modeling (Ding et al. 2014, 2015). Simultaneously, Rouskin et al. (2014) published “DMS-seq”. In DMS-seq, it is worth mentioning that, following polyA purification and size selection, an RNA fragmentation step is required for adaptor ligation and library completion. The additional RNA processing required in DMS-seq may increase the risk of RNA degradation at undesired sites. Aside from that, both methods provide information on in vivo and genome-wide RNA secondary structures with single nucleotide precision. Other advances in structurome chemistry have been to enrich for chemically modified RNAs such as by modification of NAI for pull down using click chemistry (Spitale et al. 2015).

Another approach for RNA structurome study with DMS or SHAPE probing, referred to as mutational profiling or “MaP,” has been developed by mapping the RNA covalent modifications produced by these reagents based on their promotion of misincorporation of nucleotides in the corresponding cDNA during RT under specialized conditions (Zubradt et al. 2017; Guo et al. 2020; Marinus et al. 2021; Yamagami et al. 2022). RT mutations are caused by the use of SuperScript II (SSII) or Marathon Reverse Transcriptase with Mn^2+^ instead of Mg^2+^. The use of TGIRT with Mg^2+^ also results in the incorporation of mismatch mutations in cDNA (Zubradt et al. 2017; Yang et al. 2022a). Both Marathon and TGIRT are derived from Group II intron-encoded RTs and are notable for being highly processive, thus minimizing stops at strong RNA structure. In MaP approaches, mutations (instead of “RT stops”) caused by DMS modification are used as indicators of non-base-paired positions, allowing multiple pieces of structural information to be obtained for a single read (Fig. 11A).

### Structuromes

Vicens and Kieft (2022) recently pointed out the essentiality of coupling structure prediction programs with experimental data. The first transcriptome-wide study of mRNA secondary structures (Table 1) focused on polyadenylated mRNAs from Arabidopsis seedlings under unstressed conditions (Ding et al. 2014). We obtained DMS-mediated A and C methylation signatures to guide structure prediction for over 10,000 different transcripts. Our report provided indications that mRNAs encoding stress response proteins are more structurally dynamic than those encoding housekeeping proteins (e.g. Fig. 11B), possibly enhancing the capacity for stress modulation of structure–function relationships. Our data also revealed RNA structural signatures associated with alternative polyadenylation and alternative splicing. With regard to alternative polyadenylation, DMS reactivity was low for the ∼2–15 nt upstream of the alternative polyadenylation site (i.e. from −15 to −2) and high for nucleotides from −1 to +5. This pattern suggests the presence of either RNA base pairing or protein binding upstream of the alternative polyadenylation site, and the absence of structure and protein binding at and downstream of the same site (Ding et al. 2014). With regard to alternative splicing, DMS reactivity was low for the ∼40 nt upstream of the alternative 5′-splice site for a set of unspliced transcripts. This pattern suggests that either RNA base pairing or protein binding disfavors the first step of splicing (Ding et al. 2014), which is understandable given the importance of base pairing between the 5′-exon and the spliceosome, specifically the U5 snRNAs, next to the 5′-splice site (Fig. 6B). A subsequent in vivo SHAPE analysis of Arabidopsis RNAs specifically isolated from nuclei also identified structural motifs related to RNA processing (Liu et al. 2021). When considering only these nuclear mRNAs, SHAPE reactivity was again high across the site of polyadenylation. There were also nuclear mRNA-specific marks relative to the site of polyadenylation including diminished protection in the −15 to −2 region, which could be due at least in part to the use of SHAPE (which does not report on the Watson–Crick face) rather than DMS. There was also gain of SHAPE reactivity in the −28 to −17 region, attributed by the authors to exposure of the conventional polyadenylation signal (PAS) motif. Similar to our observations on mature (polyadenylated) mRNAs (Ding et al. 2014), in the nuclear mRNA samples, SHAPE reactivity for unspliced events was low at the 5′-splice site and/or branch point (Liu et al. 2021).

From our Structure-seq dataset (Ding et al. 2014), we also identified a relationship between mRNA DMS reactivity and the structure of its translation product, wherein structured regions of the mRNA were found adjacent to sequences encoding structured regions of the protein. We speculate that this correlation between protein structure and neighboring mRNA structure may be because structured RNA regions slow ribosome processivity and thus facilitate correct folding of the preceding structured protein domains (Tang et al. 2016). An analogous relationship was reported by Weeks and colleagues for the HIV-1 RNA genome (Watts et al. 2009); its generality awaits further investigation in other biological systems.

In the prior section, we described results from early studies that employed gel-based readouts to assess the structures of individual plastid RNAs of Chlamydomonas. More recently, the structures of multiple plastid-encoded mRNAs have been studied in Arabidopsis through in vivo and in vitro structurome analysis using DMS and SHAPE probing combined with next-generation sequencing (Gawronski et al. 2021). Several key results emerged from this study. Photosystem II (PSII) is the first protein complex of light-dependent oxygenic reactions. D1 and D2 proteins are central to this complex, participating in most electron transfer activities and functioning in minimizing oxidative damage arising from photosynthesis (Nelson and Yocum 2006). D1 protein translation is increased under high light intensities, and this was found in Arabidopsis to be controlled by light-responsive RNA structural changes in the 5′-UTR of *psbA* RNA, which encodes the D1 protein (Fig. 8B) (Gawronski et al. 2021). Under high light intensity, a region around the SD sequence and a complementary region upstream of that sequence show greater reactivity to DMS and SHAPE chemical probes, and this restructuring is posited to be induced by protein binding to a distal 5′-UTR region. Resultant loss of sequestration of the SD sequence would be expected to facilitate ribosome binding, and the light-induced structural change is indeed associated with enhanced D1 translation, as assayed by ribosome profiling (Fig. 8B). By contrast, mRNAs with strong SD sequences, e.g. *rbcL* (Rubisco LSU) that were assessed, did not exhibit these light-induced changes in structure; however, similar structure changes were also observed in other plastid genes that, like *psbA*, have weak SD sequences (Gawronski et al. 2021), suggesting that this structural response represents a general but not universal mechanism of light-responsive translational regulation in the chloroplast.

For a genome-wide view of the heat-regulated RNA structurome in a plant species, we performed Structure-seq analysis on the RNA structurome of rice seedlings by in vivo DMS probing following 10 min of 42 °C heat shock (Fig. 8A) (Su et al. 2018). We observed that high temperature unfolded RNA structure, as expected from thermodynamics, and strikingly decreased the abundance of transcripts that showed signatures of unfolding/loss of protein protection, i.e. increased DMS reactivity, while those that exhibited decreased DMS reactivity under heat tended to increase in abundance. We have now observed this “inverse relationship” (Fig. 11C) between change in chemical probe reactivity and change in transcript abundance for multiple organisms and stresses: the above-mentioned heat stress in rice (Su et al. 2018), salt stress in Arabidopsis (Tack et al. 2020), heat stress impacts on tRNA unfolding in *E. coli* (Yamagami et al. 2022), and amino acid starvation in the soil bacterium *B. subtilis* (Ritchey et al. 2020), suggesting that the inverse relationship is a prevalent feature in at least 2 of the domains of life and across diverse stresses.

The inverse relationship in rice suggested that melting of mRNA structure under heat shock globally facilitates RNA degradation (Fig. 8A). This was verified by the observation of accelerated turnover of a few of these mRNAs, as detected by RT-qPCR under heat shock in the presence of a transcriptional inhibitor, cordycepin (Su et al. 2018). Of note, 3′-UTRs exhibited greater heat-induced structural unfolding than 5′-UTRs and coding sequence (CDS) regions during acute heat shock. Importantly, we found no evidence for the presence of canonical RNA thermometers known from bacteria, suggesting that the RNA response to heat in plants is largely different and utilizes the whole transcriptome as a type of thermometer. In addition, based on Ribo-seq data there was no correlation between changes in RNA structure and changes in translation induced by heat shock (Su et al. 2018); it is possible that a longer heat treatment is needed for a correlation between structural changes in the transcriptome and protein translation to emerge.

Like heat, salinity is a prevalent environmental stress that limits plant growth and severely reduces crop productivity worldwide (Hernandez 2019). To gain insight into how this common environmental stress impacts the RNA structurome in vivo, we conducted an RNA structurome study in Arabidopsis under salinity stress and compared impacts on leaves and roots (Tack et al. 2020). In our observations, salt stress conditions not only triggered alterations in RNA secondary structure globally, but the unfolded RNA transcripts also exhibited reduced transcript abundance (Figs. 8C and 11C). As we observed for heat shock (see above), we found an inverse correlation between RNA DMS accessibility, a measure of RNA unfolding and protein unbinding, and RNA abundance. This correlation was heightened in those transcripts wherein DMS accessibility changed concordantly, meaning that under salinity stress all 3 regions of the transcript (5′-UTR, CDS, and 3′-UTR) exhibited increased DMS reactivity or all 3 regions exhibited decreased DMS reactivity. Strikingly, the inverse correlation was more pronounced in tissue-specific transcripts than in tissue-shared ones (e.g. photosynthesis genes of the leaf vs. housekeeping genes of both tissues), suggesting a strong tissue-specific influence on the NaCl-responsive RNA structurome (Tack et al. 2020). Interestingly, the root and shoot RNA structuromes overall had more similar DMS reactivity under salt stress, consistent with the observation that the RNA refolding cosolutes proline, sodium, and potassium converged in concentration in the 2 tissues in the presence of NaCl.

Additionally, salt stress has been shown to promote N6-methyladenosine (m^6^A) modification of 3′-UTRs in Arabidopsis (Anderson et al. 2018). Given the dynamic nature of m^6^A deposition and its implications for the RNA-binding proteome and RNA structurome, a later study assessed the effects of m^6^A modification on RNA secondary structure and abundance (Kramer et al. 2020). After salt stress, the authors observed that 3′-UTRs that were less structured were positively correlated with the presence of m^6^A modification; however, no such correlation was seen at m^6^A sites present in non-salt-treated (control) plants, nor was protein binding correlated with m^6^A modification. The salt-induced m^6^A modifications were correlated with increased RNA stability and, thereby, proposed to enhance salt-stress-responsive protein translation. The authors report that they did not observe the inverse relationship between change in RNA structure and change in RNA abundance seen in salt stress by Tack et al. (2020) and under heat shock by Su et al. (2018). Significant differences between the studies include in vivo structure probing in Tack et al. (2020) vs. in vitro structure probing using nucleases in Kramer et al. (2020), and assessment of the entire structurome in Tack et al. (2020) vs. the nuclear structurome in Kramer et al. (2020). It is worth mentioning that upon excluding the m^6^A modified transcripts reported by Anderson et al. (2018) from our salt stress data sets, the inverse relationship between change in transcript reactivity and change in transcript abundance persists. Also, independent of RNA structure, protein binding, or m^6^A modification, similar effects of salinity on the composition of the transcriptome were observed in the 2 studies (Tack et al. 2020). Thus, m^6^A modification does not appear to be necessary to see the dominant inverse relationship between DMS accessibility and abundance.

### Long noncoding RNAs

RNAs are traditionally divided into 2 classes: “coding” for mRNA and “noncoding” (nc) for everything else. This division reflects the relatively recent recognition of the regulatory function of noncoding RNAs (ncRNAs). There are many categories of ncRNA such as the aforementioned tRNA, rRNA, snRNA, ribozymes, riboswitches, and RNA thermometers within UTRs. These particular ncRNAs are highly structured, and many of their structures have been solved by crystallography or NMR. There are many other classes of ncRNAs as well, which have been revealed by next-generation sequencing and bioinformatics, as well as a growing awareness that such RNAs exist and should be looked for. A major class of ncRNAs are small RNAs, typically 21–24 nt, which are generally not structured in their mature form. As previously mentioned, they are covered by other articles in this Focus Issue, so we do not discuss them in this review. Another class of ncRNAs are the lncRNAs, which have received increased attention over the last decade (Statello et al. 2021). These untranslated RNAs are generally longer than 200 nt and often regulate gene function. Estimates of the number of unique lncRNAs in humans range from 16,000 to 100,000. The biogenesis, subcellular localization, and function of lncRNAs are distinct from mRNAs. Long noncoding RNAs can interact with proteins, DNA, and other RNAs, and their functions are varied, including regulation of non-membranous compartments, altering the stability of and translation of mRNAs, and modulating chromatin expression. In humans, lncRNA pathways have been linked to cancer, neuronal disease, and immune response. Expression of lncRNAs appears to depend on tissue and condition, making them biomarkers and potential activators for therapeutics. Many lncRNAs in humans are transcribed by RNA Pol II, capped at the 5′-end with m7G, polyadenylated at the 3′-end, and spliced.

The structures of lncRNAs are an active area of research. One structural feature of some lncRNA is their interaction with DNA, which influences chromatin structure. Such lncRNA–DNA interactions can occur as RNA•(DNA)_2_ triplexes or as R-loops (Statello et al. 2021). Methods such as TrIP-seq are being developed to identify triplexes, and their sequences follow preferred patterns of palindromic polypyrimidine or polypurine stretches (Buske et al. 2011; Maldonado et al. 2019). In 1 example, the lncRNA *KHPS1* forms a triple helix with dsDNA in the promoter of the proto-oncogene *SPHK1*, which recruits a histone acetyltransferase, alters chromatin structure, and drives *SPKH1* expression (Postepska-Igielska et al. 2015). R-loops are recognized by proteins that can regulate gene expression both in cis and in trans. Some lncRNAs drive the formation of non-membranous compartments including the long form of *NEAT1* (Yamazaki et al. 2018), which appears to lack significant self-structure but may participate in long-range interaction between multiple lncRNAs (Lin et al. 2018). Likewise, *MALAT1* localizes to nuclear speckles and has many long-range interactions (Lu et al. 2016) including with other RNAs such as *NEAT1* and U1 snRNA, as revealed by RNA in situ conformation sequencing (RIC-seq) (Cai et al. 2020). It appears that a number of lncRNAs serve as such “RNA hubs”. Finally, it appears that some lncRNAs have important tertiary structure. For instance, *MEG3*, which is involved in the regulatory network of the tumor suppressor p53, has multiple conserved pseudoknots (Uroda et al. 2019).

Just as in mammalian systems it has become evident that the vast majority of the plant genome is transcribed into noncoding RNAs with as yet unknown functions. For instance, it is estimated based on a formidable analysis of over 16,000 RNA-seq datasets uploaded to NCBI that 4 Brassicaceae species, including Arabidopsis, harbor a combined total of approximately 130,000 long intergenic RNAs (lincRNAs), with over 20,000 in Arabidopsis alone (Palos et al. 2022). As described above for non-plant systems, the importance of RNA structure in the function of plant lncRNAs is a topic of active research. For example, R-loops induced by lncRNA have been demonstrated to play important roles in plants (Gao et al. 2021). The auxin-inducible APOLO (*AUXIN-REGULATED PROMOTER LOOP*) lncRNA recognizes multiple sites in trans via short segments leading to the formation of R-loops. This interaction has a number of effects (Ariel et al. 2020; Fonouni-Farde et al. 2022) including decoying of LIKE HETEROCHROMATIN PROTEIN 1 (LHP1; a component of the Polycomb Repressive Complex 1) away from the target genes and resolution of chromatin loops, resulting in upregulation of a suite of genes in response to auxin.

Targeted analysis of 2 Arabidopsis natural antisense lncRNAs, *COOLAIR*, involved in flowering time regulation, and *PHO1.2*, involved in phosphate nutrition, have definitively shown a role for lncRNA structure in controlling the abundance of the corresponding sense mRNA. These 2 lncRNAs are discussed in detail below.

Flowering is a fundamental stage in a plant's life cycle that generates genetic variability and allows genetic materials to be passed to the next generation. Consequently, this process offers the possibility of breeding new varieties with better agronomic characteristics to withstand environmental challenges (Pereira and Coimbra 2019). The transcription factor *FLOWERING LOCUS C* (*FLC*) represses flowering initiation and is centrally involved in vernalization-dependent flowering time regulation (Soppe et al. 2021). In 2016, Dean, Hawkes, and colleagues discovered that the natural antisense lncRNA of *FLC*, *COOLAIR*, has pivotal functions in regulating *FLC* transcription in response to vernalization (Hawkes et al. 2016). In this report, although low sequence similarity was shown in *COOLAIR* transcripts across Brassicaceae species, secondary structures were conserved. This finding implicated the importance of RNA structure and its biological functions in an evolutionary scope. Single nucleotide polymorphisms (SNPs) in *COOLAIR* among natural Arabidopsis accessions resulted in varied isoforms and distinct secondary structures that were found to be involved in tuning flowing time. For example, the haplotype of the Var2-6 accession results in a shorter H4 helix with a larger internal loop that likely decreases the stability of *COOLAIR*; this allele was less inhibitory of *FLC* transcription when introgressed into Col-0, resulting in later flowering (Li et al. 2015; Hawkes et al. 2016).

Recently, temperature-responsive *COOLAIR* RNA structural variants have been investigated through a full-length in vivo single-molecule RNA structure probing method combined with long-read PacBio sequencing (Yang et al. 2022a). *COOLAIR* processing results in class I transcripts of 400 nt, and Class II transcripts of 600–700 nt, the latter of which are upregulated by cold and have been shown to be structurally conserved (Hawkes et al. 2016). In this 2022 report, 3 structural conformations of Class II.i transcripts were detected. Two of these showed no structural alterations in response to temperature, while the third exhibited formation of a long stem between helices 4 and 6 (H4 and H6) under cold but not warm growth conditions. Mutations that strengthened this stem had no effect on *FLC* abundance or flowering time as assayed under warm conditions. However, mutations that augmented the size of a bulge in the region adjacent to H6 increased binding of Class II.i transcripts to chromatin at the *FLC* transcription start site region based on chromatin isolation by RNA purification (ChIRP) and were associated with reduced *FLC* abundance and shortened time to flower under warm conditions (Fig. 3F). Whether the mechanism involves RNA–DNA duplexes, a dsDNA–RNA triplex, or *COOLAIR* association with a regulatory protein complex remains to be elucidated. Nevertheless, these findings reveal a role of *COOLAIR* structural conformations in regulating *FLC* expression and flowering.

Inorganic phosphate (Pi) is an essential macronutrient in both plants and animals, as it is an essential building block for nucleic acids, phospholipids, various cofactors, and ATP, the energy currency of the cell. In plants, Pi modulates starch synthesis through regulating the activity of ADP-glucose pyrophosphorylase. The rice *PHOSPHATE1.2 (PHO)*-type Pi transporter (*PHO1.2*) loads Pi into the xylem for distribution via the vascular system, playing an important role in maintaining Pi homeostasis and grain production (Ma et al. 2021a). Notably, a cis-natural antisense RNA of the *PHO1.2* gene (*cis-NATPHO1.2*) in Arabidopsis has been shown to have a unique regulatory function in enhancing the translation of *PHO1.2* (Jabnoune et al. 2013; Reis et al. 2021). Pi starvation induces expression of *cis-NAT PHO1.2* RNA, which was proposed based on in vitro SHAPE-MaP studies to alter the conformation of a structured GC region located in the third exon of *PHO1.2* mRNA (Fig. 8D). The addition of *cis-NAT PHO1.2* RNA results in enhanced accessibility of *PHO1.2* mRNA to the 60S ribosome and formation of the 80S complex in an in vitro translation system and in a plant protoplast transient transfection system. Increased translation of a reporter gene fusion upon mutation of the GC region of *PHO1.2* also was observed in transient expression studies, supporting this model (Reis et al. 2021). Although additional studies in vivo are required to validate the structural aspects of this model, these results present a possible mechanism of cis-NAT translational enhancement through an RNA structure-switch mediated by sense–antisense RNA interaction.

### Pan-structuromes

Accompanying the publication of the first draft human genome sequence (Lander et al. 2001) was a second paper that reported on over 1.4 million human SNPs (Sachidanandam et al. 2001). SNPs in coding regions are designated as synonymous or nonsynonymous, with the latter but not the former causing a change in the amino acid sequence of the encoded protein. It is now appreciated that synonymous SNPs, as well as those occurring in noncoding regions such as UTRs and introns, can also be under selection; for example, synonymous mutations have the same frequency of disease association in humans as nonsynonymous mutations (Chen et al. 2010).

Among the possible functional consequences of an SNP is a change in RNA structure. The term “riboSNitch” was coined by Alain Laederach and colleagues to designate such structure-changing SNPs (Halvorsen et al. 2010). Experimental observation of a riboSNitch was actually achieved over a decade earlier, in the pre-genomic era. Vincent Stanton and colleagues (Shen et al. 1999) used nuclease probing methods to reveal that synonymous SNPs in 2 human mRNAs gave rise to different cleavage patterns at sites >10 bases away, suggesting that alleles of these mRNAs folded differently. Several other early papers applied structure probing methods to reveal riboSNitches in allelic variants of individual genes of mammals and viruses (Westerhout et al. 2005; Nackley et al. 2006; Sabarinathan et al. 2013).

The first transcriptome-wide experimental assessment of human riboSNitches was provided by Howard Chang and colleagues, who analyzed the ex vivo structuromes of a mother/father/child trio using nuclease susceptibility (Wan et al. 2014). They found that 1,907 out of 12,233 SNPs (∼15%) caused alterations of secondary structure, of which 211 were correlated with changes in transcript abundance in an eQTL database, and 22 of which were associated with human diseases. As is evident from other sections of this review, differences in RNA structure can affect transcript stability, splicing, sites of covalent modification, interaction with other macromolecules, and translation. Given that all of these processes can be affected by riboSNitches, not only variation in protein sequence and structure but also variation in RNA structure can be a target of natural selection (Halvorsen et al. 2010; Chursov et al. 2013).

With the proliferation of genome sequences, computational methods have been developed for riboSNitch prediction (Corley et al. 2015) and a database, riboSNitchDB, of over 24,000 predicted human riboSNitches is available (Lin et al. 2020). However, it is worth pointing out that the number of “gold standard” experimentally validated riboSNitches is still less than 20 (Corley et al. 2015).

**Figure 12.**
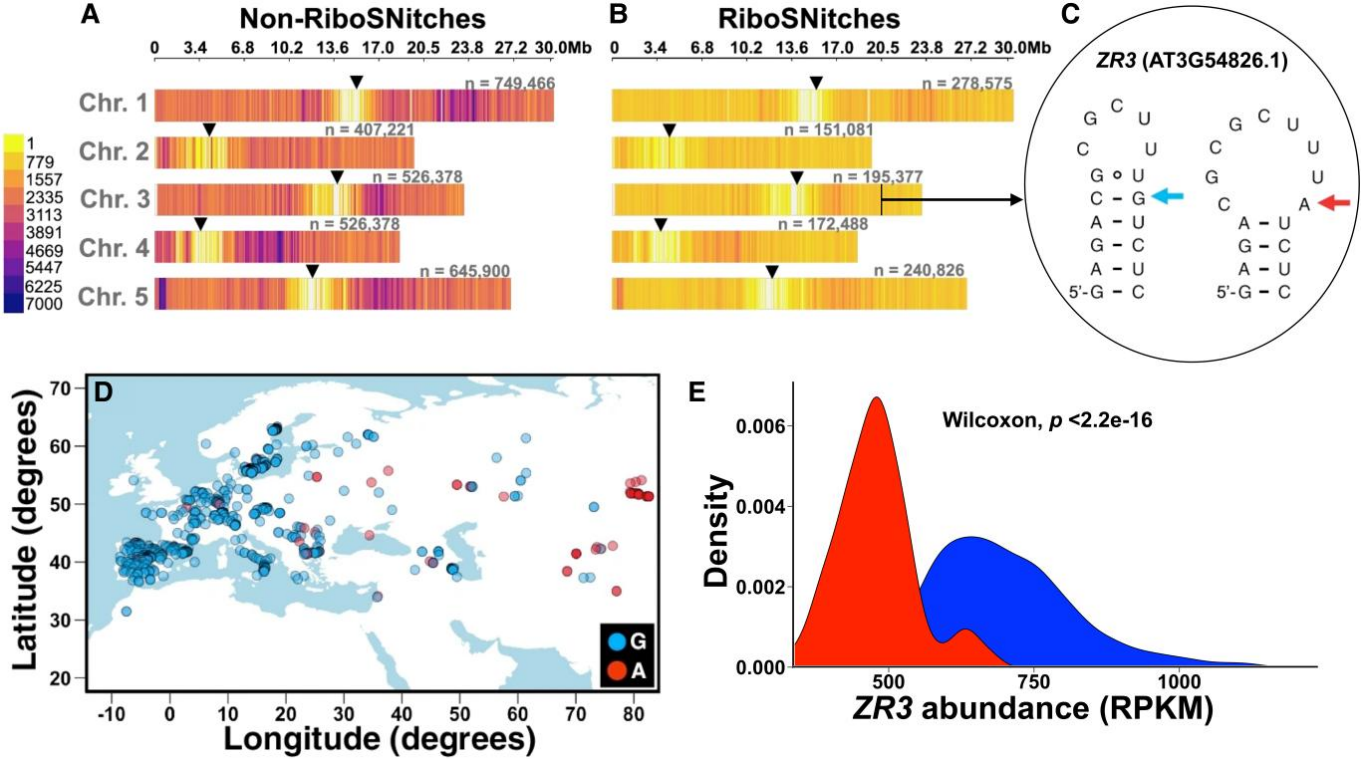
Pan-structurome prediction of riboSNitches in Arabidopsis and attributes of the *ZINC RIBBON3* (*ZR3*) Arabidopsis riboSNitch. A) Non-riboSNitches and B) RiboSNitches mapped onto the 5 chromosomes of Arabidopsis (Ferrero-Serrano et al. 2022). riboSNitches were predicted using SNPFold (Halvorsen et al. 2010). Color bars indicate the number of SNPs. C) The *ZR3* riboSNitch reference (left) and alternative (right) alleles. The destabilizing shorter stem and larger loop of the alternative allele (right) causes it to melt at lower temperatures (Ferrero-Serrano et al. 2022). D) Differential distribution of Arabidopsis accessions harboring the reference (blue dots) and alternative (red dots) across Eurasia (Ferrero-Serrano et al. 2022). E) *ZR3* transcript abundance is lower in accessions harboring the alternative allele.

Whole-genome sequencing in the plant kingdom preceded sequencing of the human genome, with the first sequence of the Arabidopsis genome reported in 2000 (Arabidopsis Initiative 2000). Interestingly, one of the first single-transcript studies of RNA structural variation in Arabidopsis was of the noncoding natural antisense transcript, *COOLAIR*, where natural variant sequences were shown to differentially affect levels of the sense transcript, *FLC* which, as discussed above, encodes a key regulator of flowering time (Hawkes et al. 2016). Arabidopsis has at least 2 advantages over mammals in the study of riboSNitches. First, Arabidopsis is selfing, and exists in nature as natural inbred lines or accessions, such that when attempting to link genotype to phenotype, there is no need to disambiguate the impact of heterozygous alleles. Second, because plants are sessile, the selective pressures they experience from the abiotic environment can be readily quantified as climate and soil parameters prevailing at the site where the accession grows. We recently created a database, CLIMtools, in which we employed GWAS approaches to identify statistically significant associations between natural variation in over 800 Arabidopsis accessions and hundreds of different climate variables; association with a given geoclimatic parameters suggests that the gene in which the associated SNP resides is involved in local adaptation to that climate conditions (Ferrero-Serrano and Assmann 2019). By applying the SNPfold algorithm to these genomes, we identified the first “pan-structurome” of a plant species. Of the approximately 3.8 million mRNA SNPs that we assessed, roughly 1 million (∼27%) are predicted to be riboSNitches (Fig. 12, A and B); thus, CLIMtools at present provides the largest database of predicted riboSNitches for any organism (Ferrero-Serrano et al. 2022).

We experimentally verified riboSNitches in 2 mRNAs, *ZINC RIBBON3* (*ZR3*) and *COTTON-GOLGI-RELATED3* (*CGR3*). Particularly for *ZR3*, the alternative structure, with its larger loop and shorter stem (Fig. 12C), melted at lower temperatures, suggesting riboSNitch conditionality dependent on temperature, a behavior that will become of increasing relevance due to global climate change. It is likely relevant that the 2 *ZR3* alleles showed statistically significant distributions across Eurasia, with the alternative (minor) allele being associated with continental regions with greater variation in temperature (Fig. 12D). The alternative allele is also associated with decreased transcript abundance relative to the reference (major) allele (Fig. 12E), suggesting that this alternative SNP may affect transcript production or turnover, possibly in a temperature-dependent (conditional) manner (Ferrero-Serrano et al. 2022). The concept of a conditional riboSNitch is also applicable in non-plant systems, considering the dynamic nature of the cellular environment.

Another interesting aspect of plant riboSNitches is that polyploid species provide whole-genome allelic differences present within each cell. For example, tetraploid durum wheat has 2 subgenomes, AA and BB. In a recent study (Yang et al. 2021), approximately half of the gene pairs between AA and BB homeologs were found to exhibit differential translation in polysome profiling. Based on in vivo SHAPE data, ∼40% of homeologs showed structural divergence, and the extent of structural divergence and the extent of differential translation were positively correlated. Transcripts with greater SHAPE reactivity, indicative of less structure and/or protein binding, tended to have higher translation rates. Of the SNPs between homeologs, 1.17% (∼3,500) were designated by the authors as riboSNitches. One riboSNitch in the 5′-UTR of the gene *TRITD2Bv1G159660* was experimentally verified to impact translation in a reporter gene assay using luciferase gene fusions with the AA vs. the BB 5′-UTR sequence. Based on the F_ST_ index of selection, the authors suggest that riboSNitches experienced greater positive selection than non-riboSNitches during wheat domestication.

## Conclusions and future directions

In this article, we reviewed the contributions of structure to the functions of venerable as well as newcomer RNAs including tRNA and its derivatives, rRNA, telomerase RNA, spliceosomal RNA, ribozymes, riboswitches, and lncRNA. We saw that the evolution of technique—from sequencing to RNA crystallography to cryoEM to next-generation sequencing methods—has played a critical role in revealing RNA structural complexity and diversity. The structure–function paradigm of RNA, established by these approaches, is modulated by covalent modifications not only to tRNA and rRNA but also to mRNA and other RNAs, as well as by the presence of GQ structures. We also saw that plants have specialized variations on these RNA classes. Regarding tRNAs, the catalog of tRFs in plants continues to grow (Zahra et al. 2022). In some cases, these are a demonstrated source of small RNAs that funnel into gene regulatory pathways. However, for many tRFs, particularly those induced by abiotic stresses, specific functions remain unknown (Ma et al. 2021b). Organellar ribosomes display characteristics of unique interacting proteins and extensions of rRNAs that distinguish the chloroplast ribosome from the mitoribosome and from the cytoplasmic ribosome. Alternative splicing in plants is distinguished by intron retention that might originate from stress interfering with intact base pairing in spliceosomal structure and, therefore, function. Other functional RNAs well known in bacteria seem to play a lesser role in plants. For instance, bacterial-type RNA thermometers, as well as some small ribozymes and almost all riboswitches, both typified by their pseudoknot-compacting structures, appear to be relatively rare and often with mysterious functions. In plants, Group II introns have lost their catalytic functions, and RNase P has lost its (catalytic) RNA altogether.

Despite the striking advances described above, the vast majority of RNAs in the vast majority of organisms remain structurally uncharacterized. Most RNAs do not adopt a globular structure, and/or are not very abundant, and so have not been crystallized. Moreover, particularly for paper-like RNAs, the functional structures adopted in vivo are likely to differ significantly from those adopted in vitro—and to be highly malleable in response to changes in cellular ionic and osmotic conditions and interactions with RNA-binding proteins and RNA-modulating enzymes such as helicases.

How RNA structure is regulated by RNA modifications is another area of intense interest. That the epitranscriptome is highly relevant to plant biology was recently revealed by laboratory-to-field studies demonstrating that transgenic expression of a mammalian RNA demethylase, FTO, increases yield in rice and potato (Yu et al. 2021). While to date attention has largely been paid to RNA modifications well known from tRNA, a recent report demonstrates functional consequences of novel glycan modification of RNAs (Flynn et al. 2021). Emerging mass spectrometric methods able to detect novel strong RNA-metabolite interactions (Rizvi et al. 2018; Rizvi and Nickbarg 2019) promise to reveal yet another layer of RNA structure regulation.

As an additional level of complexity that is layered on dynamic cellular conditions, many RNAs can adopt multiple structures, and many of these can co-exist simultaneously in vivo as structural ensembles, as recently revealed for variants of the plant lncRNA *COOLAIR,* as well as genome-wide in Human Immunodeficiency Virus I (HIV-1) RNA and SARS-CoV-2 (Tomezsko et al. 2020; Lan et al. 2022). Emerging experimental methods that utilize long-read single-molecule secondary structure readouts to enable a population-level view of RNA in vivo will greatly advance our understanding of “living” RNA. Computational advances employing deep learning methods of structure prediction, comparable to AlphaFold and Rosetta for protein (Baek et al. 2021; Jumper et al. 2021), also hold great promise, as suggested by recent advances in the prediction of RNA tertiary structure (Townshend et al. 2021).

At the level of quaternary structure, intermolecular RNA–RNA interactions can also influence, and be influenced by, structure. That RNA–RNA interactions can promote condensate formation is well established (Bevilacqua et al. 2022), yet the role of RNAs in the production and regulation of non-membranous compartments has rarely been studied in plants. Finally, at the level of quinary structure, given that under desiccating stresses (heat, drought, salinity) plants produce compatible solutes such as proline and glycine betaines that are well-known disruptors of RNA structure in the test tube (Lambert and Draper 2007), plants may offer an optimal system to study elusive effects of quinary interactions within a genuine biological context.

Finally, in an RNA-centric view of modern biology, the efficacious delivery of mRNA vaccines against SARS-CoV2 via lipid nanoparticles stands as an astounding scientific success story (Fig. 1) (Dolgin 2021). Notably, this RNA delivery mechanism is reminiscent of a mechanism by which plants combat fungal pathogens, namely via small RNAs that suppress pathogen gene expression upon their introduction into the pathogen from exocytosed plant vesicles (Wang et al. 2016; Niu et al. 2021). Whether RNA structure plays a role in the vesicular partitioning and function of these small RNAs is an intriguing question, but these parallels lead us to speculate whether vaccine developers and infectious disease specialists could learn from this plant process and vice versa. Given the broad range of abiotic and biotic conditions to which sessile plants are subject, and which are known from biophysical studies to influence RNA structure, plants are a marvelous system to reveal novel RNA structure–function relationships unanticipated or even nonexistent in other organisms.
